# Battery‐Inspired Electrochemical Synapses for Neuromorphic Applications

**DOI:** 10.1002/advs.77065

**Published:** 2026-08-18

**Authors:** Won Woo Lee, SeongCheol Jang, Wangmyung Choi, Jaewon Park, Jaehyun Hur, Hocheon Yoo, Hyun‐Suk Kim

**Affiliations:** ^1^ Department of Artificial Intelligence Semiconductor Engineering Hanyang University Seoul Republic of Korea; ^2^ Department of Energy and Materials Engineering Dongguk University Seoul Republic of Korea; ^3^ Department of Electronic Engineering Hanyang University Seoul Republic of Korea; ^4^ School of Chemical, Biological, and Battery Engineering Gachon University Gyeonggi Republic of Korea

**Keywords:** battery‐inspired, electrochemical transistors, ion‐gating, neuromorphic devices, synapse devices

## Abstract

Artificial synapses capable of analog memory and adaptive learning are key components for neuromorphic computing systems. To emulate biological synaptic functions effectively, artificial devices must exhibit gradual conductance modulation, linear responses to pulse stimuli, diverse forms of synaptic plasticity, and low‐power operation. Ion‐mediated electrochemical devices have recently emerged as promising candidates for such functionalities because ionic redistribution can continuously modify the internal electrochemical state of materials and naturally produce history‐dependent conductance changes. This review discusses recent advances in battery‐ion‐inspired synaptic devices that exploit electrochemical processes to implement artificial synaptic behavior. We first outline the fundamental electrochemical mechanisms underlying ion‐mediated state modulation, including ion insertion/accumulation, intercalation, migration, and diffusion. We then survey the materials landscape for battery‐inspired synaptic devices, covering electrolytes and active channel materials such as transition metal oxides, two‐dimensional materials, and organic mixed ionic‐electronic conductors. Emerging applications in neuromorphic computing and neuromorphic biosensing are revisited, where ionic dynamics enable the integration of sensing, memory, and computation. Key challenges related to device reliability, scalability, and environmental stability are discussed, together with future perspectives for advancing battery‐ion‐based neuromorphic technologies.

## Graphical abstract

Battery‐ion‐inspired synaptic devices integrate diverse electrolyte systems (solid, ion‐gel, liquid) with transition metal oxides, two‐dimensional materials, and organic channels to regulate ion‐electron coupling. By tailoring ion transport and host interactions, these platforms enable controllable plasticity and energy‐efficient implementations for neuromorphic computing, reservoir architectures, and intelligent sensing applications.

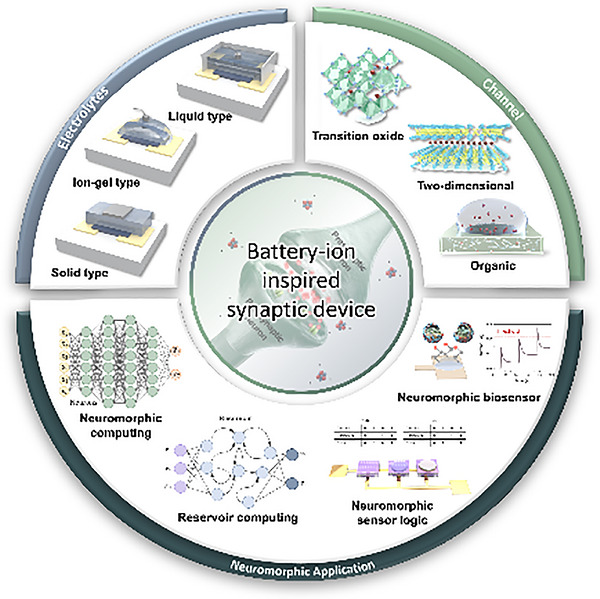

## 1 Introduction

The rapid growth of artificial intelligence has created a demand for hardware systems capable of processing information in an energy‐efficient [1, 2], adaptive [3, 4], and parallel manner [5, 6]. In contrast to conventional von Neumann architectures [7, 8], which suffer from the physical separation of memory and computation, neuromorphic computing seeks to emulate the operating principles of the human brain, where information processing is carried out through the collective dynamics of neurons [9] and synapses [10, 11]. By integrating memory and computation within the same physical elements, neuromorphic systems offer a promising route toward low‐power and intelligent hardware for next‐generation computing applications.

For neuromorphic hardware to effectively emulate biological learning processes, artificial synapses must exhibit several key characteristics, including analog memory states [12, 13, 14], gradual and linear weight updates under pulse stimulation [15, 16, 17, 18], and diverse forms of synaptic plasticity [19, 20, 21]. Multi‐level conductance modulation enables continuous representation of synaptic weights, while linear and symmetric responses to electrical pulses are critical for stable learning in hardware neural networks [22, 23, 24, 25]. As another key factor, low‐voltage and energy‐efficient operation is highly desirable. In this viewpoint, electrochemical and ion‐based devices offer significant advantages. Representative examples have demonstrated that electrochemical energy‐storage systems can also emulate synaptic functionalities with high energy efficiency. For example, Wang et al. reported a PVA/LiCl gel electrolyte‐based electrochemical supercapacitor for energy‐efficient neuromorphic operation. In this system, energy is stored and released through charging (potentiation) and discharging (depression) processes, respectively. By incorporating neuromorphic functionalities into ion‐driven electrochemical devices, the system demonstrated low energy consumption along with balanced weight update characteristics [26]. Because ionic redistribution can gradually modify the internal electrochemical state of a material, these systems naturally support analog conductance modulation [27, 28], history‐dependent responses [29, 30], and rich plasticity behaviors [31, 32, 33, 34] with a low‐voltage and energy‐efficient manner [35, 36]. Studies on ion‐coupled electronic systems and electrochemical iontronics have highlighted the potential of ion‐mediated state modulation and electrochemical gating for next‐generation electronic and neuromorphic devices [37, 38, 39]. Electrochemical switching and atomic‐switch‐type systems based on ionic migration and redox reactions have also demonstrated controllable conductance modulation and memory characteristics for neuromorphic applications [40, 41, 42]. In particular, such ion migration and redox reaction‐based switching processes have already been associated with battery effects in earlier studies of memristive devices. In 2013, Valov et al. demonstrated that redox‐based nanoionic ReRAM cells are intrinsically governed by non‐equilibrium electrochemical states, where chemical‐potential gradients generate an internal electromotive force through Nernst, diffusion, and Gibbs–Thomson potentials [43]. This internal electromotive force gives rise to nanobattery behavior and non‐zero‐crossing *I*–*V* characteristics, and can influence resistive‐switching dynamics, including filament formation/rupture, memory‐state evolution, and retention behavior. This early framework clarified that ionic redistribution and redox processes can act as internal electrochemical state variables in memristive devices, providing a basis for later artificial neurons and synapses based on ion‐mediated state modulation. Ion‐mediated devices have been considered promising platforms for implementing artificial synapses with controllable memory dynamics and energy‐efficient operation. In terms of practical applications, battery‐ion‐inspired synaptic devices offer several distinctive advantages arising from their ion‐mediated electrochemical operation. Their intrinsic ionic characteristics allow potential implementation in neuromorphic biosensing applications, while the electrochemical gating mechanism supports low‐voltage operation and gradual analog conductance modulation [2]. The reversible ion insertion and extraction processes facilitate relatively linear and symmetric depression characteristics, which are advantageous for stable synaptic weight updates in neuromorphic computing [44, 45]. However, these devices also involve inherent trade‐offs, including relatively slow switching speeds governed by ionic transport kinetics and challenges in maintaining device‐to‐device uniformity and long‐term reliability in large‐scale integrated arrays [46, 47].

**FIGURE 1:**
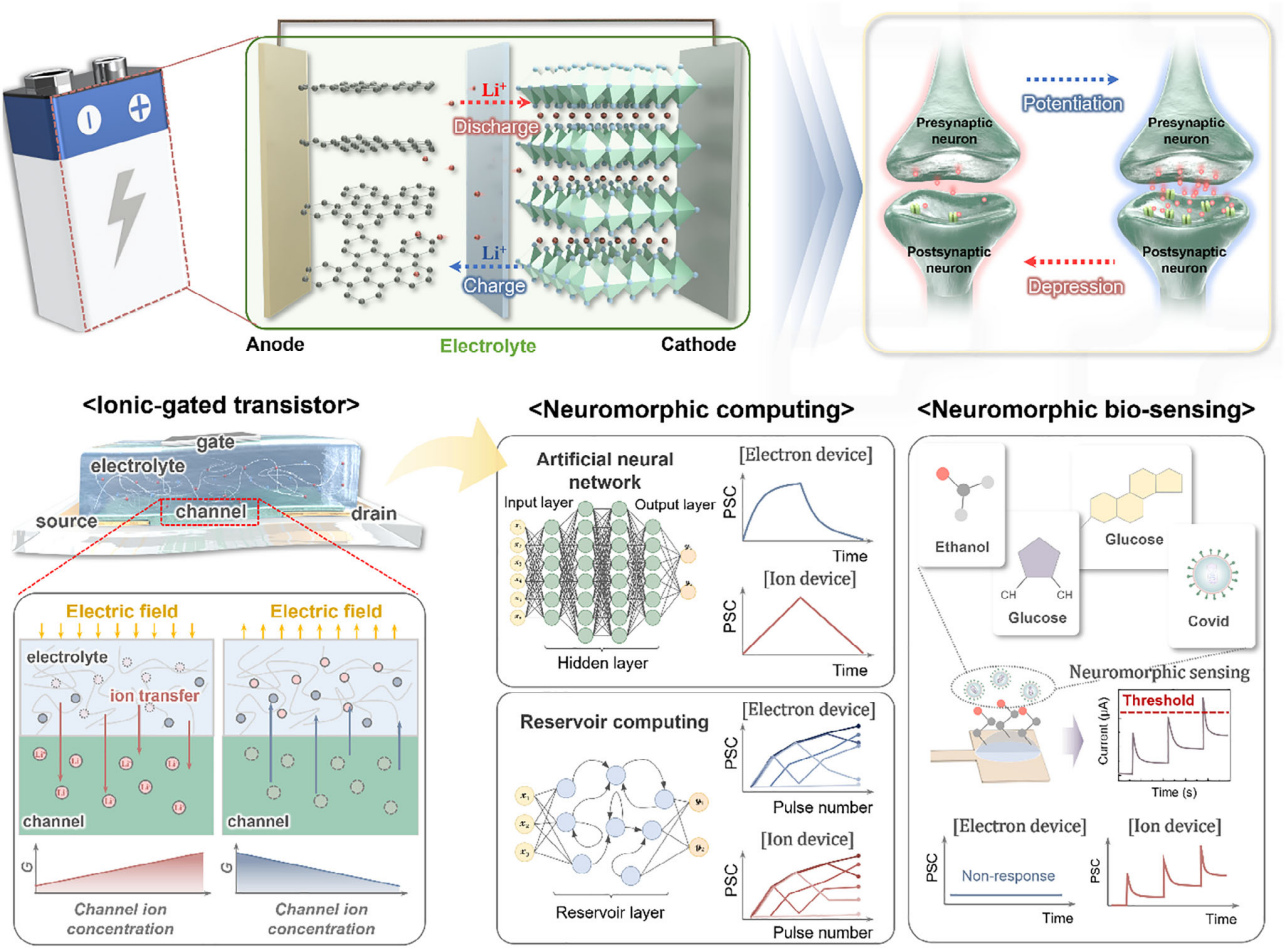
Schematic illustration of a battery‐inspired synaptic transistor for neuromorphic computing and biosensing, including the overall device overview, ion‐mediated operation mechanism, and potential neuromorphic applications such as artificial neural networks, reservoir computing, and neuromorphic biosensing.

Rechargeable batteries provide a representative example of electrochemical state evolution, where ionic motion, redox reactions, and diffusion‐driven relaxation collectively define and retain an internal state as shown in Figure 1. Among various ion‐mediated systems, rechargeable batteries provide a particularly relevant physical framework for understanding and designing electrochemical synaptic devices, as shown in Figure 1. In battery systems, electrical energy is stored and released through coordinated ionic transport and redox reactions within electrode materials [48, 49, 50]. The spatial distribution and dynamics of ions determine the electrochemical state of the system, which evolves continuously during charging and discharging processes. Interestingly, similar ion‐driven state modulation also governs the operation of many electrochemical synaptic transistors, where ionic redistribution within the device modulates channel conductance and stores synaptic weights [51, 52]. While previous studies have broadly explored ion‐mediated switching and memory phenomena in two‐terminal nanoionics and memristive devices [40, 41, 42, 43], we focus on ion‐mediated three‐terminal synaptic transistors, where ion insertion/accumulation, intercalation, migration, and diffusion can be independently and systematically regulated through a gate terminal. Such an additional control capability enables more gradual, controllable, and reproducible analog conductance modulation. In this sense, electrochemical state modulation observed in rechargeable batteries provides a useful conceptual framework for interpreting ion‐mediated conductance modulation and memory formation in neuromorphic devices. However, it is important to note that not all electrochemical synaptic devices are strictly battery‐inspired, as various architectures operate independent of bulk host intercalation. Depending on the device design, synaptic plasticity can be successfully achieved through dielectric charge trapping/detrapping enhanced by proton‐electron coupling induced by a thin electrolyte layer, or mechano‐responsive ion trap‐and‐release behavior [53, 54].

In this review, we revisit recent progress in battery‐ion‐inspired synaptic transistors and their potential for neuromorphic systems. We first introduce the fundamental electrochemical principles underlying ion‐mediated state modulation, including (i) ion insertion/accumulation, (ii) intercalation reactions, (iii) ion migration, and (iv) diffusion. We then review representative material platforms used for battery‐inspired synaptic devices, covering electrolyte systems and active channel materials that enable controllable ion‐electron coupling. Subsequently, we investigate emerging applications of these devices in neuromorphic computing and neuromorphic biosensing, where ionic dynamics provide unique opportunities for integrating sensing, memory, and computation. In the last part, we discuss the key challenges that must be addressed for large‐scale integration and practical deployment, along with future perspectives for advancing battery‐ion‐based neuromorphic technologies.

## 2 Electrochemical Fundamentals Underlying Battery‐Inspired Synaptic Devices

Rechargeable batteries operate through electrochemical processes in which electrical energy is stored and released via coordinated ionic and electronic transport. In these systems, the motion of ions and the associated redox reactions within electrode materials determines the electrochemical state of the device.

Electrochemical systems generate an electromotive force when chemical or electrochemical asymmetry exists, such as differences in ion concentration, chemical potential, redox potential, electrode composition, or electrode/electrolyte interfaces [55, 56]. Rechargeable batteries are representative engineered electrochemical systems that convert chemical or electrochemical asymmetry into reversible energy storage through ion transport, electron transport, interfacial charge transfer, and redox reactions. In a simplified form, oxidation and reduction can be expressed as M → M^z+^ + ze^−^ and M^z+^ + ze^−^ → M, respectively. For example, Li extraction from a layered transition‐metal oxide cathode can be represented as LiMO_2_ → Li_1−x_MO_2_ + xLi^+^ + xe^−^, where the oxidation state of the transition metal changes to compensate the extracted Li ions and electrons. Ion redistribution in electrochemical materials is driven by both concentration gradients and electric fields and can be described by the Nernst‐Planck relation,

$$J_{i}=-D_{i}\nabla C_{i}-\left(\frac{z_{i}D_{i}F}{RT}\right)C_{i}\nabla \phi$$

where J_i_, D_i_, C_i_, z_i_, F, and ϕ represent ionic flux, diffusion coefficient, ion concentration, ionic charge, Faraday constant, and electric potential, respectively [57]. The first term describes diffusion, while the second term describes electric‐field‐driven migration. When the electric‐field contribution is negligible, ion redistribution can be approximated by Fick's second law:

$$\frac{\partial C}{\partial t}=D\frac{\partial^{2}C}{\partial x^{2}}$$

Here, C, D, x, and t represent ion concentration, diffusion coefficient, position, and time, respectively [58, 59]. The electrochemical processes that underlie rechargeable battery operation, including ion insertion/accumulation, intercalation reactions, ionic transport dynamics, and interfacial electrochemistry, provide an important conceptual framework for understanding electrochemical synaptic transistors.

In electrochemical synaptic devices, electrochemical principles are exploited to regulate channel conductance through ion migration and redistribution under an applied electrical stimulus, similar to a battery system. Different from conventional electronic devices that rely solely on electronic charge transport, electrochemical synaptic transistors utilize the motion and accumulation of ions to establish internal electrochemical states that modulate electronic transport properties. These ion‐mediated processes allow the device conductance to evolve gradually in response to electrical stimulation, providing a physical basis for analog synaptic weight updates in neuromorphic hardware.

**FIGURE 2:**
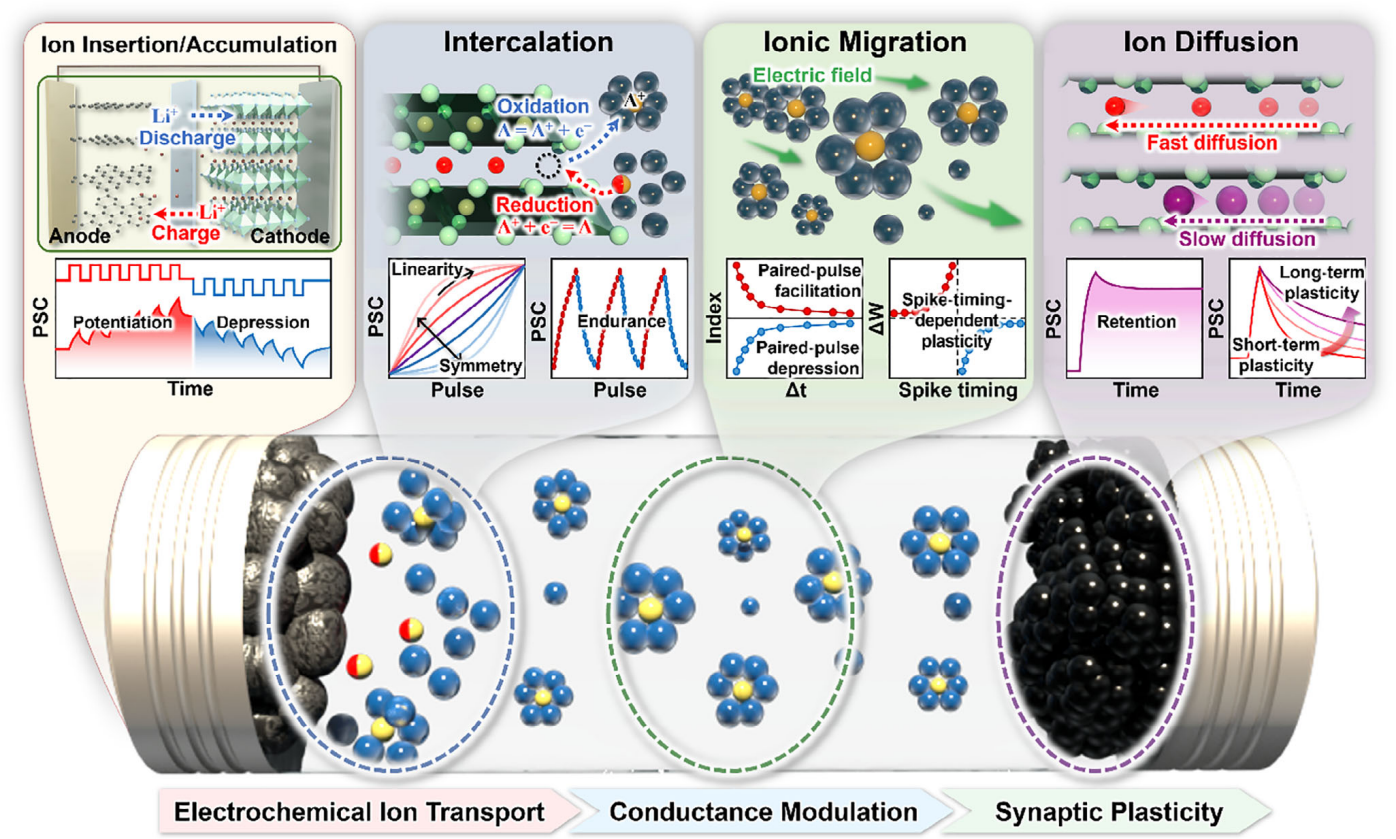
Schematic of ionic mechanisms for conductance modulation and synaptic plasticity in ion‐mediated neuromorphic devices. Four representative processes are illustrated: 1) ion insertion/accumulation, involving the accumulation and release of ions for reversible conductance modulation; 2) Intercalation, where redox‐coupled ion insertion and extraction enable gradual conductance changes; 3) Ionic migration, an electric field‐driven drift facilitating temporal learning behaviors (PPF, PPD, STDP); and 4) Ion diffusion, a gradient‐driven transport governing relaxation dynamics. Diffusion kinetics, dictated by intrinsic ionic properties, strongly influence retention behavior and the transition between short‐ and long‐term plasticity.

This section discusses the fundamental electrochemical processes that enable ion‐mediated state modulation in both electrochemical energy storage systems and electrochemical synaptic transistors (Figure 2). Understanding these mechanisms provides a unified perspective on the way ionic dynamics can serve as internal state variables that encode information in both energy storage systems and neuromorphic devices.

### 2.1 Ion Insertion/Accumulation

Ion insertion/accumulation is similar to potentiation/depression because the electrochemical state of a rechargeable battery during charging and discharging is determined and maintained by the concentration and distribution of ions within the electrode materials [60]. In electrochemical synaptic devices, potentiation generally occurs through ion insertion or accumulation within the active material, resulting in increased conductance states and the storage or retention of synaptic information. Conversely, depression is associated with ion redistribution, extraction, or relaxation processes, leading to decreased conductance and gradual forgetting or erasure of stored synaptic information [61, 62]. Although these processes fundamentally differ from rechargeable batteries in terms of practical energy storage and delivery, the reversible ionic dynamics underlying potentiation and depression remain highly analogous to the charging and discharging processes in electrochemical energy‐storage systems. The electrochemical state of the electrode is therefore determined by the concentration and distribution of ions inside the active material, which is commonly described as the state of charge (SOC) in rechargeable battery systems [41, 42].

Because ionic content evolves continuously during electrochemical operation, the electrochemical state of the system changes gradually rather than abruptly. This continuous evolution enables batteries to store and release energy in a controllable and reversible manner. Importantly, ionic configurations established during electrochemical operation can remain stable even after removal of the external electrical stimulus, allowing rechargeable batteries to maintain stored energy over repeated charge‐discharge cycles [63, 64].

A similar principle applies to electrochemical synaptic transistors. In these devices, electrical signals are used to program and read an internal ionic configuration that determines the conductance state of the channel [65, 66, 67]. The stored ionic distribution therefore acts as an internal state variable that regulates electronic transport through the device. The conductance state of an electrochemical synaptic transistor reflects the accumulated history of ionic motion and electrochemical reactions within the device, enabling ion‐controlled modulation of electronic transport.

### 2.2 Intercalation

Intercalation is associated with analog weight updates through the reversible insertion and extraction of mobile ions within a host structure under electrical stimulation, enabling continuous control of ionic concentration within the active material [55, 68, 69, 70]. Ions occupy available interstitial sites within the host lattice while the overall crystal framework remains largely preserved. Such structural stability enables repeated ion insertion and extraction and is typically accompanied by reversible redox reactions within the host lattice. To maintain charge neutrality, the oxidation state of transition‐metal ions changes during ion insertion and extraction [71, 72, 73]. As a result, modulation of the electronic structure and charge density continuously tunes the conductance state of the material, thereby enabling gradual and reversible synaptic weight updates [12]. Linearity and symmetry are commonly used metrics to evaluate the quality of analog weight updates during potentiation and depression processes.

Reversible intercalation is governed by the relationship between the mobile ion and the host structure. In addition to ionic size, ionic charge, electrolyte environment, accessible diffusion pathways, lattice strain, ion‐host interaction, and redox compatibility determine whether ion insertion and extraction can proceed reversibly [68, 74]. Small monovalent ions such as H^+^ and Li^+^ can participate in reversible insertion or electrochemical doping in various host materials, including layered, tunnel‐type, and open‐framework structures, when suitable transport pathways and charge‐compensation mechanisms are available [75, 76, 77]. In contrast, larger monovalent ions such as Na^+^ and K^+^ can experience limited available space or lattice deformation in compact host structures, while multivalent ions such as Mg^2+^ and Ca^2+^ often show reduced transport due to stronger electrostatic interactions with the host framework [78, 79, 80]. Therefore, ion intercalation in electrochemical synaptic devices should be understood as a host‐dependent process governed by both ionic properties and host‐lattice characteristics.

The coupling between ionic occupancy and electronic structure offers an important role in electrochemical synaptic transistors. When ions migrate into the channel material, they can interact with the lattice through electrochemical doping, interfacial charge accumulation, defect modulation, or reversible redox processes that modify carrier density and transport properties [12, 81]. Since the extent of ion insertion can be gradually controlled by electrical stimulation, intercalation‐driven electrochemical doping provides a natural mechanism for analog conductance modulation in neuromorphic devices.

### 2.3 Ion Migration

[R4Q2] Ion migration involves the transport of ions through an electrolyte under an electric field and their subsequent redistribution near electrodes or within active materials in electrochemical systems [49, 64, 82]. As mobile ions redistribute within the electrochemical system, the local electrochemical state evolves over time and influences subsequent responses to electrical stimuli [76]. Electrochemical synaptic transistors similarly rely on migration‐driven ionic transport mechanisms to regulate channel conductance. In these devices, ionic redistribution induced by electrical stimulation governs the modulation of electronic transport within the channel [83, 84, 85]. The speed and extent of ion migration determine how rapidly the conductance state can be updated in response to electrical pulses. When consecutive voltage pulses are applied within short temporal intervals, remaining ionic redistribution produced by a preceding pulse can influence the response to subsequent stimuli. This interaction leads to characteristic synaptic behaviors such as paired‐pulse facilitation (PPF) or paired‐pulse depression (PPD), where the amplitude of the second response depends on the temporal spacing between input pulses [86]. Ion migration dynamics can also give rise to spike‐timing‐dependent plasticity (STDP), in which the relative timing between pre‐ and postsynaptic spikes determines the direction and magnitude of conductance updates [33, 87, 88].

### 2.4 Ion Diffusion

Ion diffusion involves the spatial redistribution and relaxation of ionic species within electrochemical materials following electrical stimulation. As ionic configurations gradually evolve toward equilibrium through diffusion‐driven processes, the retention and temporal stability of electrochemical states are determined [89]. Electrochemical synaptic transistors similarly rely on diffusion‐driven ionic relaxation processes following electrical stimulation. Because ionic motion is significantly slower than electronic transport, ionic configurations generated during programming do not immediately return to equilibrium after removal of the external bias [90]. Instead, these ionic configurations gradually relax through diffusion‐driven processes, leading to time‐dependent conductance evolution in synaptic devices.

This diffusion‐driven relaxation process regulates the retention characteristics of synaptic conductance states. When ionic redistribution relaxes rapidly, conductance changes decay over short time scales, giving rise to short‐term plasticity (STP). In contrast, deeper ionic penetration or energetically stabilized ionic configurations can maintain conductance states for extended periods, resulting in long‐term plasticity (LTP). Therefore, ion diffusion governs the temporal stability of ionic configurations and plays a crucial role in controlling the transition between short‐term and long‐term memory behaviors in electrochemical neuromorphic devices [91, 92].

## 3 Materials Landscape for Battery‐Inspired Synapses

### 3.1 Electrolytes

**FIGURE 3:**
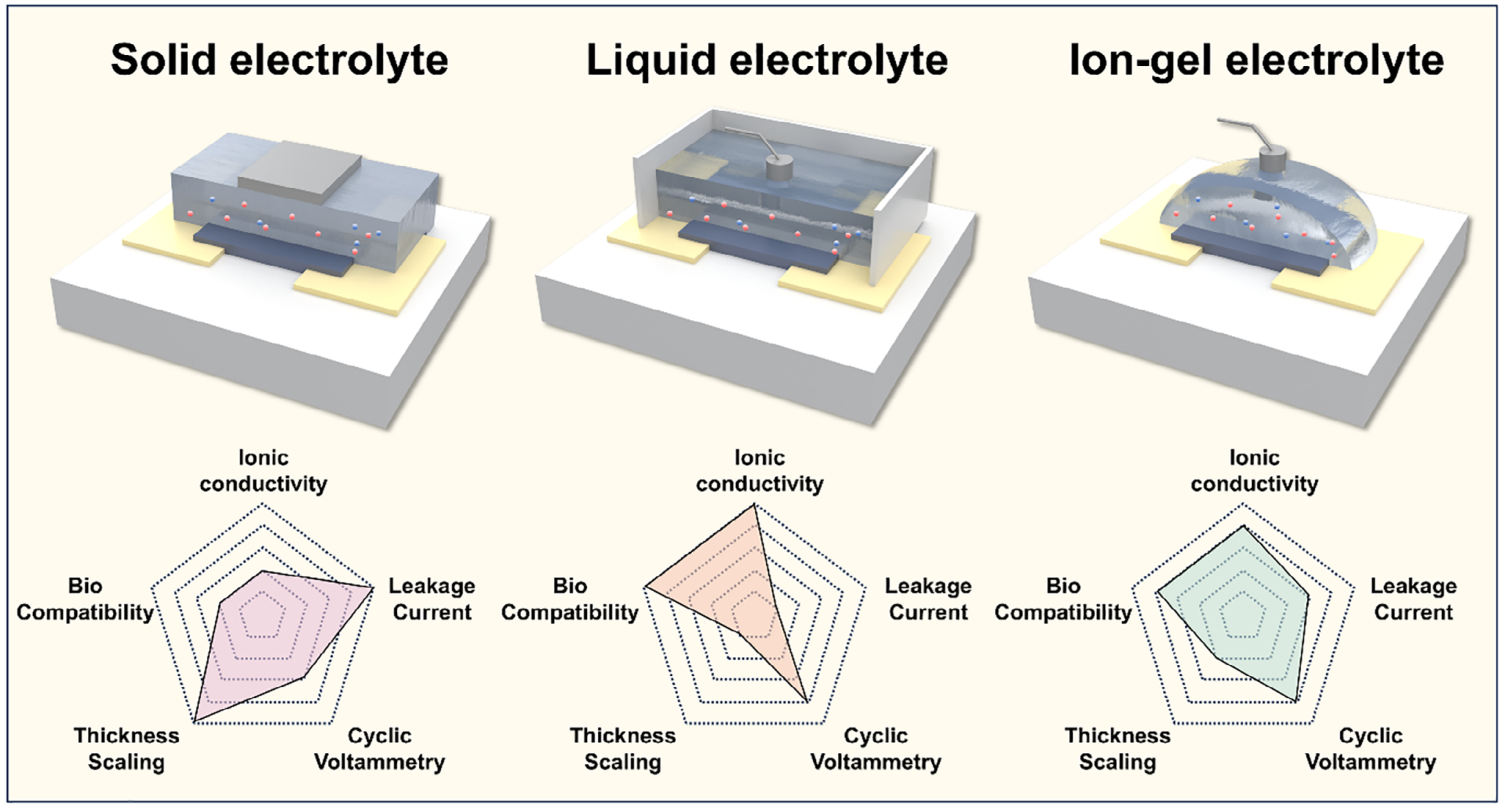
Performance trade‐offs according to electrolyte type in battery‐inspired synaptic devices. The radar charts show the distinct advantages of each electrolyte system. Ion‐gel electrolytes provide a balanced profile between processability and ionic transport, whereas solid‐state electrolytes are more suitable for high‐density integration despite lower ionic conductivity. Liquid electrolytes remain the gold standard for fundamental mechanism studies due to their exceptional ion transport.

Electrolytes contribute as a crucial key on battery‐inspired synaptic transistors by simultaneously serving as the ion reservoir, the transport medium, and the interface that governs ion‐channel interactions. Rather than solid intercalation hosts, which operate under well‐defined crystallographic constraints, electrolytes span a wide range of physical forms, including solids, liquid, and ion‐gels [93, 94, 95, 96, 97]. As shown in Figure 3, each electrolyte class exhibits distinct ionic mobility, solvation environment, and interfacial behavior. These differences critically influence the device response to electrical bias, determining the kinetics, retention, depth of conductance modulation, and ultimately whether the resulting plasticity corresponds to short‐term or long‐term learning.

In general, ionic conduction in electrolytes can be understood as a thermally activated process that follows the Arrhenius law:

$$\sigma \left(T\right)=\sigma_{0}\exp\left(-\frac{E_{a}}{kT}\right)$$

where *σ*(T) is the ionic conductivity, *σ_0_* is the pre‐exponential factor associated with the concentration and mobility of charge carriers, *E_a_* is the activation energy for ion migration, *k* is the Boltzmann constant, and *T* is the absolute temperature [98, 99, 100, 101]. This relationship indicates that electrolyte performance is governed not only by the number of mobile ions, but also by the activation energy associated with ionic transport. In many electrolyte systems, this activation energy reflects both the energetic cost of generating mobile ionic defects and the migration barrier that ions must overcome, which is strongly influenced by the local bonding environment and the connectivity of available hopping sites within the lattice. Electrolyte materials with higher mobile‐ion availability and lower migration barriers can induce faster ionic redistribution under electrical bias, leading to faster switching and larger conductance modulation.

#### 3.1.1 Solid Type

Solid electrolytes provide a structurally robust platform for battery‐inspired synaptic transistors, offering well‐defined interfaces and strong compatibility with semiconductor fabrication processes [102, 103]. Their ionic conductivity is typically lower than that of liquid‐based systems because ion transport is constrained by the solid lattice. Consequently, ionic motion occurs over longer timescales, which often translates into slower but more predictable conductance modulation. This characteristic is advantageous for achieving stable synaptic states and suppressing excessive short‐term fluctuations. For example, Liu et al. demonstrated that ion transport through a LiPON electrolyte enables stable synaptic functions such as excitatory postsynaptic current and paired‐pulse facilitation in a Se_0.3_Te_0.7_ synaptic transistor [83]. Under a negative gate bias, Li^+^ ions rapidly accumulate at the semiconductor/electrolyte interface, resulting in nonlinear conductance responses. The solid‐state lattice provides defined pathways for ionic diffusion, enabling time‐dependent conductance modulation. These regulated ionic diffusion pathways offer stable and reproducible short‐term memory characteristics, demonstrating the potential of solid electrolytes for temporal information processing.

A key benefit of solid electrolytes lies in their intrinsically low leakage current and structural stability compared to liquid or ion‐gel systems. Owing to their dense and immobile solid‐state framework, undesired ionic diffusion and parasitic electrochemical reactions are effectively suppressed, enabling stable operation with minimal standby power consumption. Unlike liquid or gel‐based electrolytes, solid electrolytes do not suffer from solvent evaporation, ion redistribution, or leakage‐related instability, which contributes to improved device reliability. Furthermore, solid electrolytes can be formed as uniform and ultra‐thin films with thicknesses comparable to conventional dielectric layers, allowing compact device architectures and precise control over the ion‐transport pathway. Their thin‐film nature facilitates vertical stacking and planar device scaling while maintaining mechanical integrity. From a device operation perspective, solid electrolytes generally exhibit limited electrochemical activity under typical operating biases, reflecting restricted ion mobility and reduced interfacial charge‐transfer reactions with the channel [104, 105]. As a result, conductance modulation tends to proceed in a gradual and well‐controlled manner, favoring stable and analog‐like synaptic plasticity. However, this limited ionic dynamic represents a trade‐off, as it can limit the maximum switching speed and the diversity of synaptic responses compared to liquid or ion‐gel electrolyte systems. To address this challenge, Park et al. utilized a NASICON‐type Li_1‐x_Al_x_Ti_2‐x_(PO_4_)_3_ (LATP) electrolyte with a highly ordered crystalline structure to enhance synaptic devices exhibiting higher ionic conductivities of ∼10^−4^ S∙cm^−1^ [2]. The proposed device enabled strong electrolyte gating and wider hysteresis windows, while the crystallized LATP electrolyte contributed to improved linearity, recognition accuracy, and stable and reproducible repeated measurements. These results suggest that enhancing the crystallinity of solid electrolytes can improve ionic conductivity and gating efficiency, refine analog synaptic behaviors, and expand the design versatility of solid electrolyte‐based neuromorphic systems.

Solid electrolytes employed in synaptic transistors can be broadly categorized into inorganic solid electrolytes, organic solid electrolytes, and organic‐inorganic composite solid electrolytes. Inorganic solid electrolytes are distinguished by their high electrochemical stability and wide effective voltage windows, which enable operation under comparatively large gate fields without severe parasitic reactions. This stability is advantageous for achieving conductance states with long retention, making inorganic solid electrolytes well suited for synaptic devices that emphasize nonvolatile or quasi‐nonvolatile weight storage, analogous to LTP. In contrast, organic polymer solid electrolytes exhibit fundamentally different ion transport characteristics. Ion migration in polymer electrolytes is related to polymer relaxation and free‐volume fluctuations, resulting in ionic diffusion rather than transport through fixed sites [106, 107]. However, ionically conductive organic solid polymers are often dissolved in organic solvents or damaged during photolithography processes, limiting their compatibility with conventional device fabrication procedures. In 2025, Chen et al. proposed a polymer solid electrolyte‐gated organic synaptic transistor utilizing a photo‐cross‐linked nitrile butadiene rubber network embedded with lithium bis(trifluoromethanesulfonyl)imide (LiTFSI), enabling photolithography‐compatible solid electrolyte patterning and stable synaptic operation [108]. Li^+^ diffusion within the solid electrolyte induced significant current hysteresis and enabled synaptic‐like learning and memory behavior. The photo‐cross‐linked polymer electrolyte provided stable solid‐state ion transport pathways and reliable electrolyte‐gating operation, enabling reproducible synaptic switching characteristics under repeated operation. These results suggest that polymer‐based solid electrolytes can provide controllable ionic transport and stable analog synaptic behaviors suitable for low‐voltage neuromorphic operation.

#### 3.1.2 Liquid Type

Liquid electrolytes provide a highly dynamic ionic environment for synaptic transistors, characterized by intrinsically high ionic conductivity and rapid ion transport. As ions are solvated and free to migrate within the liquid phase, ionic conductivities are typically several orders of magnitude higher than those of solid electrolytes, enabling fast ion redistribution under an applied gate bias [109, 110, 111, 112]. As a result, liquid electrolyte‐gated synaptic transistors exhibit rapid conductance modulation, pronounced hysteresis, and strong short‐term responses, which are well suited for emulating volatile synaptic behaviors [12, 113]. Electrolyte‐gated transistors can be operated through two representative mechanisms. EDL‐based electrostatic modulation and electrochemical doping [114, 115, 116]. For EDL‐driven operation, ions accumulate at the electrolyte/channel interface without penetrating into the semiconductor channel, forming an ultrathin EDL with extremely high interfacial capacitance. By contrast, electrochemical doping involves ion penetration into the active layer, resulting in volumetric conductance modulation and persistent conductance charges. During ion penetration into the active layer, the relative contribution of EDL formation and electrochemical doping are influenced by factors such as the solvent content of the electrolyte layer [115] and the magnitude of the applied gate voltage [116]. These coupled ionic processes can complicate the interpretation of synaptic conductance modulation and retention characteristics. To distinguish and characterize these mechanisms, systematic characterization approaches including electrical characterization under different ambient conditions [115], gate‐voltage dependent absorption analysis of the active channel [116], in situ crystal structure characterization [117], and frequency dependent impedance spectroscopy [118] provide useful strategies for clarifying coupled ion modulation behaviors in electrolyte‐gated systems.

In addition to their dynamic ionic behavior, liquid electrolytes offer inherent advantages for bio‐interfaced neuromorphic systems. Their aqueous or solvent‐based composition closely resembles physiological environments, allowing efficient coupling with biological ions such as Na^+^, K^+^, and Cl^−^ [119, 120]. This bio‐mimetic ionic environment facilitates seamless integration with living tissues and enables direct transduction of biochemical or electrophysiological signals into conductance changes within the synaptic transistor. Furthermore, the soft and conformable nature of liquid electrolytes supports intimate contact with biological substrates, making them particularly attractive for neuromorphic biosensing and real‐time adaptive bioelectronic interfaces.

However, their fluidic nature introduces inherent limitations in structural stability and device integration. Reliable encapsulation is required to prevent leakage and evaporation, which can otherwise compromise long‐term operation and reproducibility. In addition, solvent evaporation, unintended ion redistribution, and electrochemical side reactions under prolonged bias stress may lead to performance drift and device‐to‐device variability. These stability concerns present challenges for large‐scale integration and extended operational reliability compared to solid‐state systems [121, 122, 123]. From a synaptic plasticity perspective, liquid electrolytes generally enable a wide range of dynamic behaviors due to their strong electrochemical activity and facile ion‐channel interactions. Conductance modulation can be abrupt and highly nonlinear, supporting diverse learning rules but often at the expense of predictability and reproducibility. Compared with solid electrolytes, liquid systems tend to favor fast, volatile plasticity rather than gradual and stable weight updates, with an emphasis on a fundamental trade‐off between speed and retention.

In a related liquid ion‐gated transistor architecture, WO_3_‐based devices were shown to emulate synaptic plasticity behaviors, including excitatory postsynaptic current, PPF, and LTP and LTD, arising from ion‐driven modulation of the WO_3_ channel conductivity [124]. These results highlight ion‐driven conductance modulation in oxide channels and its relevance to ion‐gated synaptic transistor operation. For bio‐interfaced neuromorphic systems, Liu et al. demonstrated a liquid electrolyte‐gated transistor (EGT) that directly utilizes physiological K^+^ and Cl^−^ ions to emulate synaptic plasticity under biologically relevant conditions [125]. In this system, the migration and redistribution of K^+^ and Cl^−^ ions within an aqueous electrolyte dynamically modulate the channel conductance through EDL formation and ion–electron coupling at the electrolyte–channel interface. This architecture enables direct transduction of biological ionic signals into analog conductance states, presenting liquid electrolyte‐gated synaptic behaviors for real‐time neuromorphic biosensing and adaptive bioelectronic interfaces.

#### 3.1.3 Ion‐Gel Type

Ion‐gel electrolytes are hybrid electrolyte systems composed of immobilized ionic liquids within a supporting matrix, enabling simultaneous ionic transport and mechanical stability. This structure preserves continuous ion‐transport pathways while suppressing macroscopic fluidity, enabling stable electrolyte gating without leakage or flow‐related instability [126, 127, 128]. In synaptic transistors, ion‐gel electrolytes enable efficient ion migration under an applied gate bias and drive conductance modulation via coupled ionic and electronic processes at the electrolyte‐channel interface. Owing to their high interfacial capacitance, ion‐gel‐gated devices typically operate at very low voltages and exhibit large conductance modulation [129, 130, 131]. At the same time, confinement of ions within the polymer matrix slows down ionic relaxation compared to liquid electrolytes, allowing access to both volatile and semi‐nonvolatile conductance states depending on the bias conditions.

In synaptic transistors, these characteristics are reflected in ion‐mediated conductance modulation governed by coupled ionic and electronic processes at and near the electrolyte‐channel interface. Ion‐gel‐gated devices typically operate at low voltages and exhibit large, gradual changes in channel conductance. Partial confinement of ions within the matrix slows ionic relaxation relative to liquid electrolytes, enabling bias‐dependent access to volatile and semi‐nonvolatile conductance states. Thus, synaptic weight updates can arise from interfacial charge accumulation, ion penetration into the channel or interfacial region, and reversible electrochemical processes, supporting analog‐like modulation and tunable transitions between short‐term and long‐term plasticity.

From an integration perspective, ion‐gel electrolytes exhibit practical characteristics associated with their quasi‐solid nature. They can be deposited using solution‐based processes such as spin coating or printing, allowing thickness scaling over a wide range while maintaining continuous ionic pathways [132, 133, 134]. Such thickness tunability enables control over ionic resistance, interfacial capacitance, and operating voltage, which is relevant for device optimization and circuit‐level integration. Ion‐gel layers can conformally cover planar channels and be encapsulated using conventional passivation layers, facilitating compatibility with standard device architectures. However, their gel‐like nature imposes limitations in terms of thermal budget, solvent compatibility, long‐term chemical stability, and mechanical robustness compared to fully inorganic solid electrolytes. In electrochemical characterization, ion‐gel‐based devices often exhibit finite capacitive currents and bias‐dependent responses in cyclic voltammetry, reflecting coupled ionic polarization and interfacial electrochemical activity rather than purely ideal electrostatic behavior. These characteristics, while advantageous for synaptic functionality, can complicate process integration and long‐term reliability under repeated ion‐driven or electrochemical operations.

Despite these integration and reliability challenges, ion‐gel electrolytes offer attractive platforms for neuromorphic devices because their ion‐mediated operation resembles biological synaptic signaling mechanisms. In 2024, Zhang et al. reported synaptic devices gated by a sodium alginate‐based ionic gel, in which Na^+^ ion migration within the polymeric electrolyte enables global regulation of channel conductance [135]. In these devices, the formation and relaxation of EDL and partial ionic penetration into the channel induced gradual and reversible conductance modulation, allowing the emulation of synaptic behaviors such as excitatory postsynaptic current, PPF, and learning‐forgetting characteristics. These results demonstrate that biopolymer‐derived ionic gels can serve as effective ionically active gating media for globally coupled synaptic regulation.

### 3.2 Active Layer Materials

**FIGURE 4:**
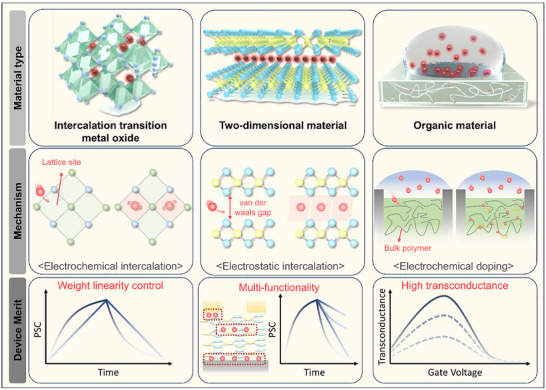
Classification of channel materials and their corresponding ion‐modulation mechanisms for tailored synaptic performance. (left) transition metal oxides (TMOs) utilize electrochemical intercalation into deterministic lattice sites, which allows for precise weight linearity control essential for high‐accuracy neural network training; (center) 2D materials leverage electrostatic intercalation within van der Waals gaps to achieve multi‐functionality by tuning layer‐specific electronic states; and (right) organic materials (OECTs) facilitate electrochemical doping throughout the polymer bulk, yielding high transconductance and superior signal amplification.

The active layer offers a decisive role in battery‐ion‐inspired synaptic transistors as it defines how ionic motion is coupled to electronic transport and ultimately determines the nature of synaptic weight modulation [124, 136, 137]. Different from conventional field‐effect devices where conductance is primarily governed by electrostatic charge accumulation at an interface, ion‐based neuromorphic transistors rely on materials that can accommodate, confine, or redistribute mobile ions in a controllable way. Depending on the host material, ions may intercalate into well‐defined crystallographic frameworks, migrate through van der Waals gaps, or penetrate into the bulk of mixed ionic‐electronic conductors. As shown in Figure 4, channel materials can be broadly categorized into three representative platforms: intercalation‐type transition metal oxides (TMOs), two‐dimensional (2D) materials, and organic mixed ionic and electronic conductors. TMOs provide deterministic lattice sites for electrochemical intercalation, enabling precise control over weight linearity and stable state retention. In contrast, 2D materials leverage ion modulation within van der Waals gaps or at atomically thin interfaces, offering layer‐sensitive electronic tuning and multi‐functional operation. Organic materials, particularly in organic electrochemical transistors (OECTs), allow volumetric ion penetration and bulk electrochemical doping, resulting in high transconductance and strong signal amplification.

#### 3.2.1 Intercalation Transition Metal Oxide‐Based Active Layers

Intercalation‐type TMO constitute one of the most mature and widely studied families of ion‐hosting materials, originating primarily from their long‐standing role as electrodes in Li‐ion batteries [138, 139, 140, 141]. Their ability to incorporate ions into well‐defined crystallographic frameworks without major structural collapse is central to their suitability for battery‐inspired synaptic devices. In these materials, ions occupy interstitial or vacancy sites such as octahedral or tetrahedral voids, and the electronic structure adjusts continuously to maintain charge neutrality. This coupling between ionic occupancy and electronic configuration is the fundamental reason why TMOs enable smooth, analog modulation of conductivity.

The core physical mechanism arises from redox‐active transition metals whose d‐electron manifolds adjust their oxidation states as ions move in and out of the host structure. When Li^+^ is inserted, electrons localize into specific cation states or into hybridized M‐O orbitals; when Li^+^ is removed, the electron population decreases correspondingly [142, 143]. This dynamic reshaping of the electronic density of states directly influences carrier concentration, band alignment, and conductivity. Because these electronic changes evolve continuously with ionic fraction (x in Li_x_MO_2_, for example), the material naturally supports a graded set of conductance states rather than abrupt switching. This behavior is essential for implementing multi‐level synaptic weights.

Crystallographically, intercalation oxides span layered structures, such as LiCoO_2_ (LCO) and Li(Ni,Mn,Co)O_2_ (NMC), spinel oxides including LiMn_2_O_4_ (LMO), and tunnel‐type hosts such as V_2_O_5_. Each structure provides distinct diffusion pathways and energetics for ionic movement [144, 145, 146, 147]. Layered oxides permit two‐dimensional migration through interslab spaces; spinels offer three‐dimensional pathways with corner‐sharing octahedra; and tunnel oxides provide open channels along crystallographic axes [148, 149, 150]. These structural motifs determine whether ion insertion is shallow and fast or deep and slower, directly influencing the timescales of synaptic plasticity achievable in a device.

When integrated into synaptic transistors, the intercalation reaction functions as the principal mechanism of conductance modulation. Under an applied gate bias, ions migrate from the electrolyte to the oxide channel and intercalate into accessible sites. Near‐surface insertion produces modest but reversible changes in local electronic charge density, enabling transient adjustments that resemble STP. Deeper insertion alters the internal bonding environment and electronic landscape, creating more persistent conductance states aligned with LTP and LTD. The degree of intercalation is tuned by the magnitude, duration, and sequence of gate pulses, allowing the device to encode stimulus history through the evolving ionic profile.

**FIGURE 5:**
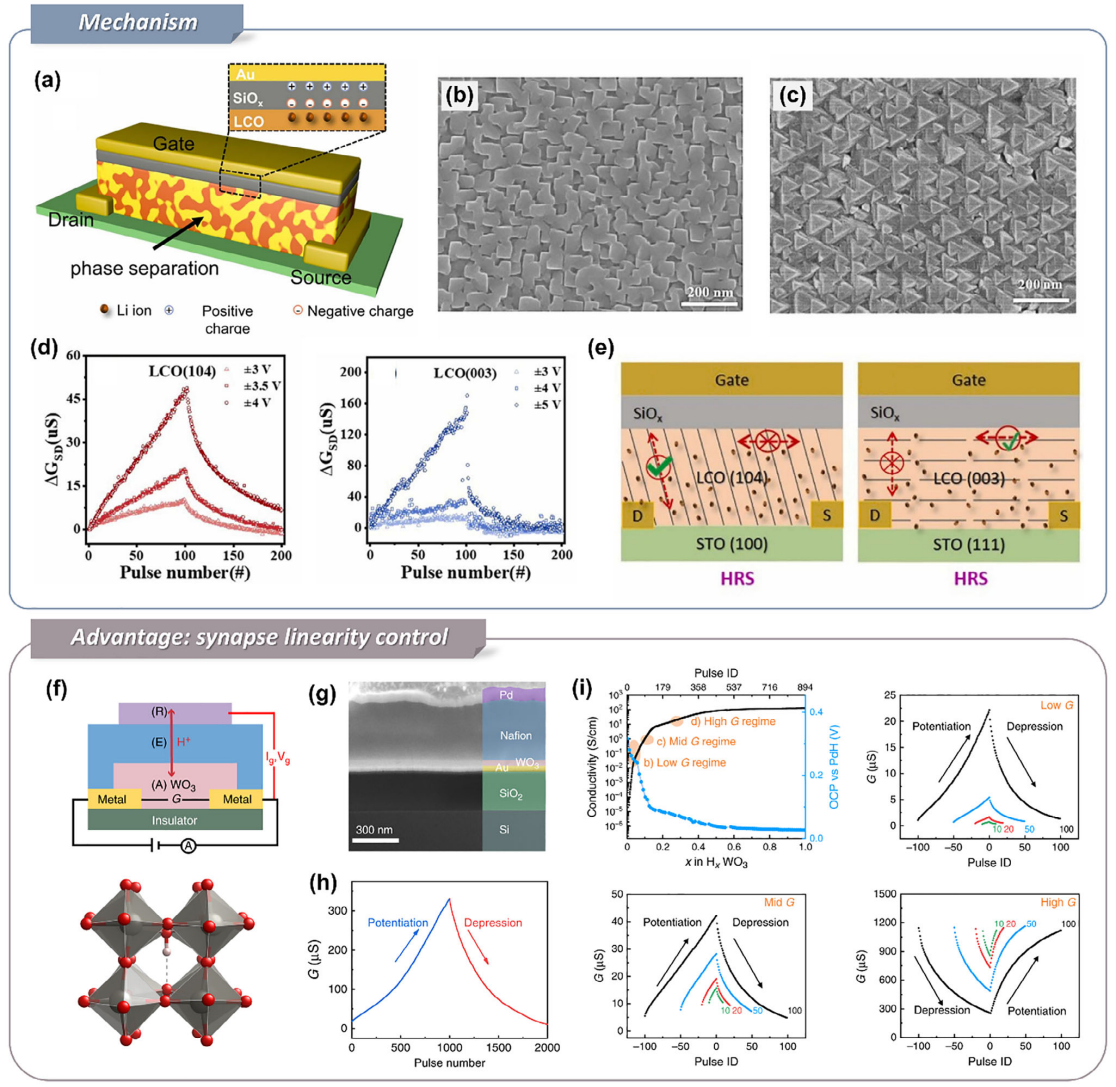
Mechanism and advantages of synaptic behavior in intercalation transition metal oxide‐based active layer transistors. (a) Schematic of an electrochemically gated synaptic transistor employing a LCO channel. (b, c) Surface morphologies of differently oriented LCO thin films. (d) Pulse‐induced conductance evolution (∆G) for LCO films with (104) and (003) orientations. (e) Schematic comparison of Li^+^ diffusion pathways in (104)‐ and (003)‐oriented LCO, highlighting how crystallographic alignment governs insertion depth and effective transport length, thereby determining synaptic weight update characteristics. Reproduced with permission [151]. 2023, Elsevier. (f) Device structure of a proton‐intercalation WO_3_ synaptic transistor. (g) Cross‐sectional image confirming the layered architecture that enables bulk proton penetration into the WO_3_ lattice. (h) Gradual and reversible conductance modulation under repeated potentiation and depression pulses. (i) Conductance‐regime‐dependent weight update characteristics and ionic transport properties, demonstrating that intermediate conductance states provide improved linearity and symmetry compared with low or high conductance regimes. Reproduced with permission [75]. 2020, Springer Nature.

Intercalation‐type oxide materials such as LCO and Li_x_V_2_O_5_ (LVO) have been employed as electrochemically active layers in synaptic transistors, where electronic charge transport is strongly modulated by ion intercalation. These materials have been extensively developed in the field of lithium‐ion batteries and are designed to accommodate reversible ion insertion and extraction within well‐defined crystallographic structures. Ion intercalation proceeds through the bulk of the electrochemically active layer and follows crystallographically defined pathways that are intrinsic to the host lattice. In particular, the LCO‐based synaptic transistor reported by Zhang et al. highlights that ion insertion is not only governed by the host chemistry but also strongly dependent on crystallographic orientation (Figure 5a) [151]. As illustrated in Figure 5b,c, differently oriented LCO films such as (104) and (003) planes provide distinct Li^+^ diffusion pathways and surface accessibility. In the (003)‐oriented film, Li^+^ ions preferentially migrate along the layered interslab direction parallel to the c‐axis, enabling more direct and energetically favorable intercalation into octahedral sites. In contrast, the (104) orientation exposes a different atomic arrangement at the electrolyte interface, leading to altered insertion kinetics and effective diffusion length. This orientation‐dependent ion transport results in markedly different conductance evolution under identical pulse stimuli (Figure 5d). The device with a favorable diffusion pathway exhibits larger and more gradual ∆G accumulation with pulse number, corresponding to smooth potentiation behavior, whereas the other orientation shows relatively limited or slower conductance modulation (Figure 5e). Consequently, synaptic weight update characteristics such as dynamic range, linearity, and symmetry are intrinsically linked to crystallographic control of ion insertion depth and pathway. These results demonstrate that, beyond material selection, crystallographic engineering provides an additional degree of freedom for tailoring analog weight modulation in intercalation‐based synaptic devices.

Another approach for improving the synaptic characteristics of ion intercalation‐driven electrolyte‐gated transistors is compositional modification. Mg‐doped LCO active layers have been demonstrated to exhibit an enhanced hysteresis window and more linearly increasing conductance modulation during Li^+^ insertion and extraction processes compared to pristine LCO [152]. These results suggest that doping engineering can effectively enhance analog synaptic characteristics by tuning the electrochemical and electronic properties of battery electrode materials. However, the driving voltage required for p‐type LCO‐based electrolyte‐gated synaptic transistors can cause degradation of the Li^+^ ion source layer during repeated operation. To address this challenge, n‐type Li^+^ ion storage materials have been explored as alternative electrochemically active layers. For example, spinel Li_4_Ti_5_O_12_ exhibits substantial conductivity modulation through reversible lithiation and delithiation processes at relatively low operating voltages compared to LCO [153]. These studies highlight that both compositional engineering and material selection are critical design strategies for ion intercalation‐driven electrolyte‐gated synaptic transistors, enabling enhanced analog synaptic characteristics, reduced operating voltage, improved operational stability, and higher energy efficiency through the tailored electrochemical and ion‐storage properties of intercalation host materials.

Other oxide materials, such as WO_3_ and MoO_3_, represent a distinct class of electrochemically active layers in synaptic transistors, characterized by their ability to accommodate ion insertion while retaining appreciable electronic conductivity. These transition‐metal oxides possess open or layered crystal structures that allow mobile ions to penetrate beyond the surface and occupy interstitial sites within the lattice under an applied bias [75, 91, 154]. Ion insertion is not confined to a single stoichiometric redox process but can proceed over a broad range of insertion depths and local coordination environments within the oxide lattice. Such flexible intercalation behavior induces changes in the electronic structure and carrier distribution of the material, resulting in conductance modulation governed by coupled ionic and electronic transport. This intercalation‐mediated response typically leads to gradual and partially persistent conductance evolution, enabling synaptic transistors to support a wide spectrum of synaptic functions spanning short‐term and long‐term plasticity.

Using intercalation mechanisms, Yao et al. reported a proton‐intercalation WO_3_ synaptic transistor, where the smaller ionic radius and faster diffusion of protons compared to Li^+^ enable systematic modulation of synaptic weights through crystallographic control of ion‐transport pathways [75]. As schematically illustrated in Figure 5f, protons are injected into the WO_3_ channel under gate bias and form H_x_WO_3_ via a reversible redox process, thereby modulating the carrier density of the oxide channel. Cross‐sectional analysis in Figure 5g confirms the well‐defined layered device structure that enables efficient proton migration into the bulk lattice rather than confinement at the interface. Because ion insertion proceeds into the interior of the WO_3_ lattice, conductance evolves gradually with repeated pulse stimulation, as shown in Figure 5h. The continuous increase and decrease in conductance during potentiation and depression indicate analog synaptic weight updates with a broad dynamic range. Importantly, the gradual conductance evolution arises from diffusion‐controlled intercalation, which distributes ions over multiple lattice sites and suppresses abrupt switching behavior. By controlling ion transport kinetics and insertion depth within transition‐metal oxides such as WO_3_, synaptic weight modulation can be optimized for reliable analog neuromorphic computation. More critically, Figure 5i reveals that the conductance update profile strongly depends on the conductance regime, reflecting differences in H^+^ ion insertion depth and diffusion accessibility. In the low conductance regime, conductance modulation is limited due to insufficient carrier accumulation, whereas in the high conductance regime the response tends to saturate. In contrast, the intermediate regime exhibits more balanced and progressive conductance change under both potentiation and depression pulses, leading to improved linearity and symmetry of synaptic weight updates. Hence, synaptic behaviors in these systems are characterized by nonvolatile or strongly persistent conductance states, making them well suited for emulating long‐term plasticity and memory retention.

#### 3.2.2 2D Material‐Based Active Layers

2D materials have emerged as a compelling active channel platform for battery‐ion inspired synaptic transistors, owing to their atomically thin geometry and highly exposed electronic states. Representative 2D semiconductors used in synaptic devices include transition metal dichalcogenides such as MoS_2_, WS_2_, MoSe_2_, and WSe_2_, as well as layered materials such as black phosphorus and graphene derivatives [155, 156, 157, 158, 159, 160]. These materials offer diverse band structures, carrier polarities, and defect landscapes, providing a versatile materials space for tailoring ion‐electronic coupling in neuromorphic transistors. Ions can be introduced and confined at multiple strategic locations, including the semiconductor dielectric interface, the van der Waals gaps between layers, grain boundaries, or beneath the source and drain electrodes [21, 161, 162, 163]. Such configurational freedom enables diverse synaptic mechanisms within a single material system, ranging from volatile electrostatic gating to semi‐nonvolatile ion trapping and long‐term memory effects. Importantly, the absence of dangling bonds and the weak interlayer bonding in van der Waals materials reduce structural degradation during repeated ion migration, supporting stable and reversible synaptic operation.

These characteristics position 2D materials as a powerful platform for neuromorphic devices in which ionic processes are not merely parasitic effects but are deliberately harnessed as key state variables. In the following sections, representative studies are discussed to illustrate how battery‐ion‐inspired synaptic transistors based on 2D materials exploit ion‐mediated interactions to achieve rich synaptic dynamics and multifunctional neuromorphic behavior.

**FIGURE 6:**
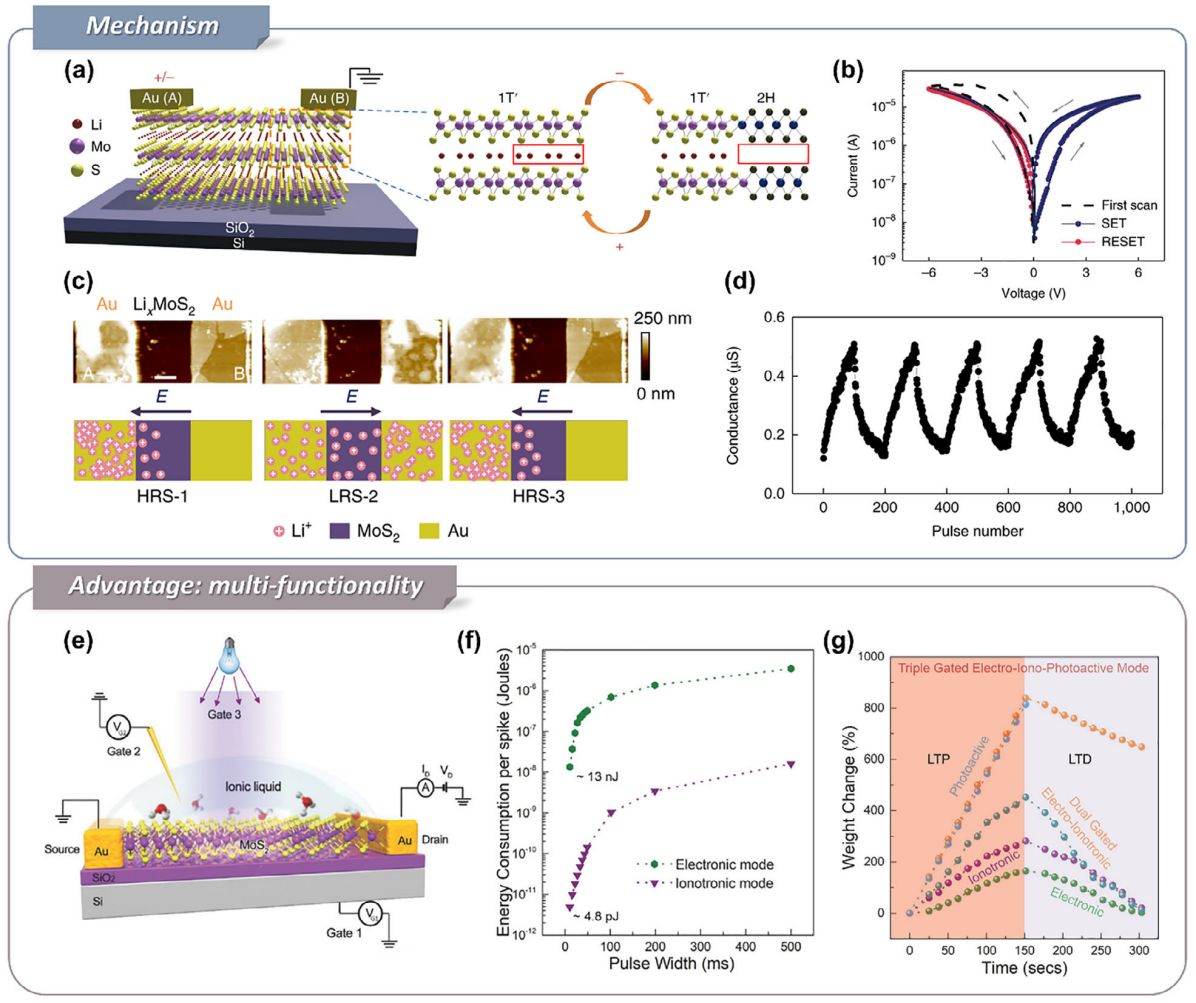
Mechanism and advantages of synaptic behavior in 2D material‐based active layer transistors. (a) Schematic of a Li^+^‐driven MoS_2_ synaptic transistor, where lateral electric fields induce Li^+^ intercalation and a reversible 2H‐1T’ phase transition within a confined channel region. (b) Hysteretic *I*–*V* characteristics arising from reversible Li^+^ insertion/extraction, indicating nonvolatile ion‐mediated conductance modulation. (c) Spatially localized Li^+^ accumulation beneath asymmetric electrodes, enabling multiple resistance states depending on ionic distribution. (d) Analog and repeatable conductance updates under pulsed stimuli, reflecting ion‐stabilized phase modulation. Reproduced with permission [164]. 2019, Springer Nature. (e) Schematic of a triple‐gated MoS_2_ device integrating electrostatic, ionic, and photoactive gating within an atomically thin channel. (f) Energy consumption per spike for electronic and ionotronic modes. (g) Analog weight modulation under electrical and photo‐assisted operation, demonstrating synergistic electronic, ionic, photonic gating. Reproduced with permission [165]. 2018, John Wiley and Sons.

Li^+^ ion migration in a 2D MoS_2_ channel has been utilized to realize an electrochemical ion‐inspired synaptic device capable of reversibly and spatially confining electronic phase modulation through ionic insertion [164]. In this work, Li ions are electrochemically driven into multilayer MoS_2_ under lateral electric fields applied between Au electrodes, forming a Li_x_MoS_2_ channel region (Figure 6a). Rather than relying on conventional electrostatic gating, synaptic behavior emerges from the controlled redistribution of Li ions within the van der Waals layered structure of MoS_2_, enabling persistent conductance modulation associated with ion‐stabilized phase transitions. A defining feature of this system lies in the unique ion interaction pathways enabled by the 2D nature of MoS_2_. Owing to its layered crystal structure and weak interlayer van der Waals bonding, Li ions can be reversibly inserted between MoS_2_ layers without catastrophic lattice disruption. This ion insertion locally induces a phase transition from the semiconducting 2H phase to the metallic 1T prime phase, as schematically illustrated in Figure 6a. Importantly, the spatial extent of the phase‐transformed region is governed by the local ionic concentration and electric field distribution, allowing analog and position‐dependent modulation of the channel conductance rather than abrupt filamentary switching.

The electrical characteristics shown in Figure 6b reveal clear hysteretic current‐voltage behavior associated with reversible Li ion insertion and extraction, highlighting the nonvolatile yet controllable nature of the ion‐induced conductance states. Complementary surface morphology and schematic analysis in Figure 6c further illustrate that Li ion accumulation can be selectively stabilized beneath specific electrode regions, leading to multiple high‐resistance and low‐resistance states depending on ion distribution. This behavior directly maps ionic history onto electronic transport, enabling gradual conductance evolution and cumulative inhibition or potentiation under repeated electrical stimuli. As demonstrated by the pulse‐dependent conductance modulation in Figure 6d, the Li ion driven phase modulation in MoS_2_ supports repeatable and analog synaptic weight updates over extended cycling. In contrast to bulk battery materials, where ion intercalation proceeds throughout a thick active layer, the atomic‐scale thickness of MoS_2_ confines ionic effects to a narrow spatial region, resulting in enhanced sensitivity and lower energy cost per switching event. This work highlights a key advantage of 2D materials in battery‐ion inspired synaptic transistors: ions are not merely charge dopants, but active agents that can reversibly reconfigure the electronic phase landscape of the channel. Such ion phase coupling is difficult to realize in conventional three‐dimensional semiconductors, underscoring the unique potential of 2D materials for neuromorphic devices that exploit ion‐mediated state evolution as a functional mechanism rather than a parasitic effect.

In contrast to the Li ion‐induced phase transition mechanism demonstrated in MoS_2_ based synaptic devices, John et al. reported a 2D chalcogenide device that exploits synergistic electrostatic, ionic, and photo‐induced effects to realize rich neuromorphic functionality [165]. This work employs layered MoS_2_ as the active channel material, but rather than relying on bulk ion insertion and structural phase transformation, synaptic behavior arises from the coexistence and coupling of multiple gating modalities acting on the atomically thin channel (Figure 6e). As illustrated in Figure 6e, the device architecture enables simultaneous electrostatic gating, ion‐mediated modulation, and optical excitation, all of which interact strongly with the electronic states of the 2D MoS_2_ channel. The atomic thickness of the material ensures that these external stimuli are not spatially separated but instead overlap within the same active region, leading to highly nonlinear and history‐dependent conductance responses. Unlike the previous Li intercalation‐based approach, where synaptic states are stabilized by ion‐induced phase changes, the conductance modulation here is governed by reversible charge trapping, ionic screening, and photo generated carrier dynamics at the channel interface.

Figure 6f shows the energy consumption of the device, where the iontronic mode (4.8 nJ per event) exhibits lower energy consumption compared to the electronic mode (13 nJ per event). Energy efficiency remains a critical challenge for artificial synapses. The reduced energy consumption observed in the iontronic mode indicates the potential of ion‐mediated synaptic devices for energy‐efficient neuromorphic computing. Furthermore, the ability to control both iontronic and electronic modes highlights the multifunctionality of the 2D material platform, providing a versatile approach for tunable device operation and adaptive neuromorphic functionalities. The neuromorphic potential of this synergistic gating concept is further illustrated in Figure 6g, where repeated electrical and optical stimuli produce analog and accumulative conductance modulation characteristic of synaptic weight updates. The coexistence of electrical, ionic, and photonic degrees of freedom allows the device to operate as a multifunctional device, capable of integrating diverse input modalities within a single 2D channel. This work underscores a complementary advantage of 2D materials in battery‐ion‐inspired synaptic transistors: beyond serving as hosts for ion insertion and phase transitions, 2D channels can act as highly sensitive platforms where ionic, electronic, and optical processes are intrinsically entangled.

These two studies highlight distinct yet complementary roles of 2D materials in ion‐based neuromorphic devices. While Li intercalation in MoS_2_ leverages structural phase reconfiguration to achieve robust and nonvolatile synaptic states, synergistic electro‐iono‐photo gating emphasizes dynamic and reversible modulation driven by interfacial ionic processes. This contrast illustrates the versatility of 2D materials as neuromorphic platforms, where the location and nature of ion involvement can be deliberately engineered to balance stability, speed, and functional richness.

#### 3.2.3 Organic‐Based Active Layer

Organic semiconductors constitute an important class of active materials for battery‐ion‐based synaptic transistors, particularly in OECT architectures. In battery‐ion and ion‐driven synaptic devices, organic semiconductors have been widely employed, with representative examples including conducting polymers such as poly(3,4‐ethylenedioxythiophene):polystyrene sulfonate (PEDOT:PSS), poly(3‐hexylthiophene) (P3HT), and diketopyrrolopyrrole (DPP)‐based polymers, as well as small‐molecule semiconductors such as pentacene derivatives [166, 167, 168, 169, 170]. Unlike conventional field‐effect transistors, where charge modulation is largely confined to the channel‐dielectric interface, OECTs operate through volumetric electrochemical doping and dedoping of the organic channel [171, 172]. Under an applied gate bias, mobile ions penetrate the bulk of the organic semiconductor, modulating the carrier density throughout the entire channel volume. This volumetric gating mechanism gives rise to a large effective capacitance and enables substantial conductance modulation even at low gate voltages. As a result, OECTs typically exhibit exceptionally high transconductance, making them highly attractive for low‐power neuromorphic computing and bio‐interfacing applications [173, 174, 175].

From a materials and processing perspective, organic semiconductors offer additional advantages that distinguish them from inorganic counterparts. Many organic channels can be deposited using solution‐based techniques such as spin coating, printing, or spray coating, enabling simple fabrication, low thermal budgets, and compatibility with large‐area and flexible substrates [176, 177, 178, 179]. The mechanical compliance and softness of organic materials further facilitate conformal integration with flexible electronics and biological systems, which is particularly relevant for wearable and implantable neuromorphic devices. In synaptic transistor applications, the mixed ionic‐electronic conduction inherent to organic semiconductors enables strong coupling between ion dynamics and electronic transport. Gradual ion penetration, accumulation, and relaxation within the organic channel naturally give rise to analog conductance modulation, temporal memory, and history‐dependent responses, which are essential for emulating synaptic plasticity. Depending on the mobility of ions and the stability of the doped states, organic synaptic transistors can access a wide spectrum of plasticity behaviors ranging from short‐term to long‐term memory.

**FIGURE 7:**
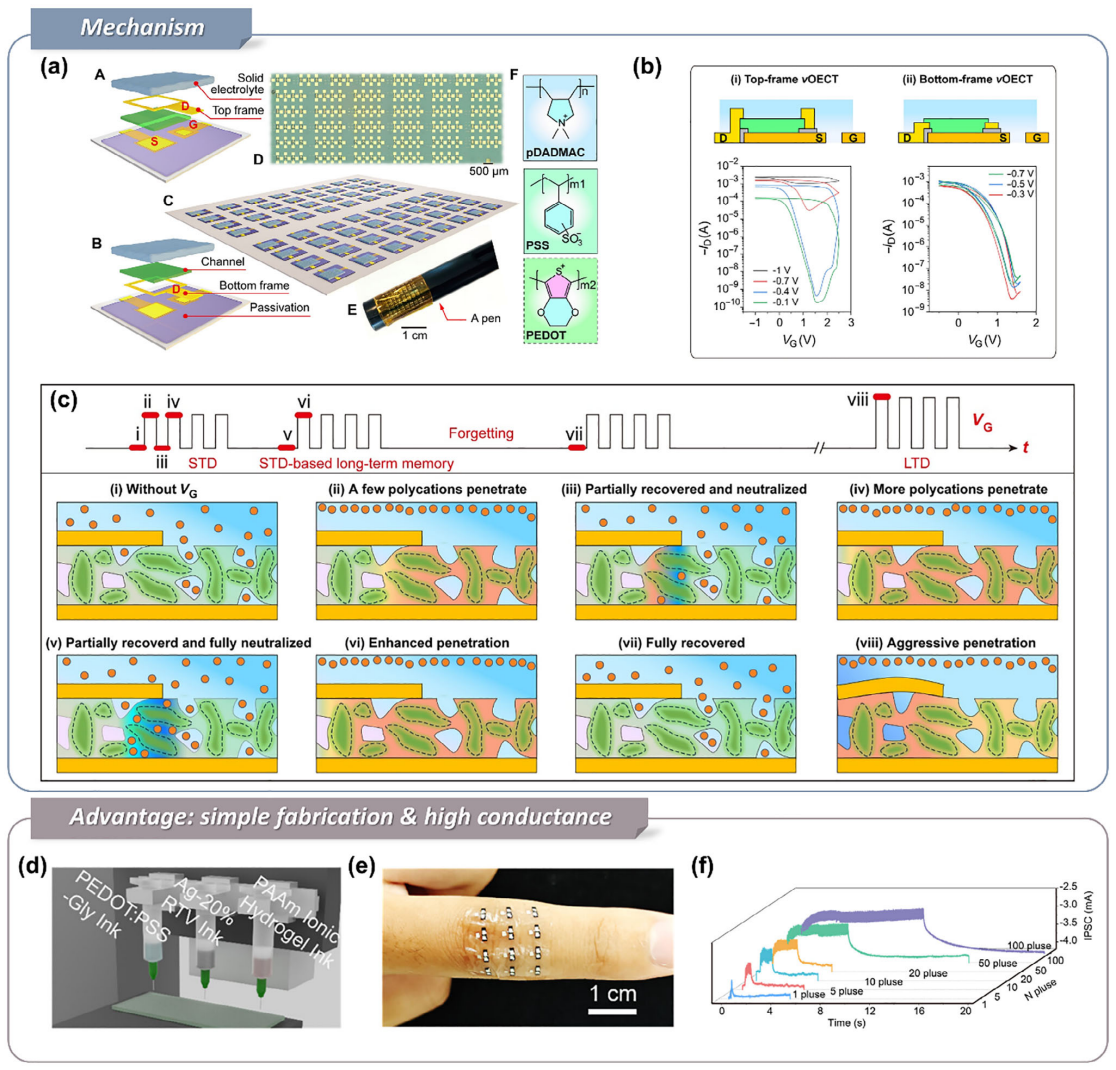
Mechanism and advantages of synaptic behavior in organic‐based active layer transistors. (a) Schematic of a vertical PEDOT:PSS OECT incorporating a solid polyelectrolyte (pDADMAC) and drain‐frame electrodes positioned above or below the channel. (b) Transfer characteristics of top‐frame and bottom‐frame configurations, showing distinct hysteresis behaviors arising from different ionic transport and confinement pathways. (c) Schematic illustration of spike‐induced ion penetration and relaxation processes in the PEDOT:PSS channel, highlighting incomplete polycation back‐diffusion and cumulative synaptic effects governed by ionic trapping. Reproduced with permission [174]. 2025, The American Association for the Advancement of Science. (d) Multimaterial 3D printing strategy for stretchable OECT arrays, integrating elastic electrodes, PEDOT:PSS channels, solid‐state electrolytes, and encapsulation layers. (e) Image of the stretchable OECT array conformally mounted on skin, demonstrating mechanical compliance and large‐area integration. (f) Synaptic conductance modulation under pulsed gate operation in the stretchable device, showing stable analog weight updates under mechanical deformation. Reproduced with permission [180]. 2023, American Chemical Society.

Device architecture and electrode engineering provide an effective approach for regulating ion‐electron coupling in polymer‐based synaptic transistors. Vertical OECTs based on a PEDOT:PSS channel and a solid‐state poly(diallyldimethylammonium chloride) (pDADMAC) polyelectrolyte demonstrated that introduction of a drain‐frame electrode structure can effectively modulate ion transport pathways and relaxation dynamics [174]. As illustrated in Figure 7a, the device incorporates drain frames positioned either above or below the organic channel, while the corresponding transfer characteristics (Figure 7b) reveal pronounced differences in hysteresis depending on the drain‐frame location. This behavior originates from the drain frame acting as an effective ionic barrier, which alters the penetration and relaxation of polycations within the PEDOT:PSS channel. Consequently, the spatial configuration of the drain electrode directly governs the balance between ionic accumulation and electronic conduction, leading to distinct synaptic response profiles. Figure 7c elucidates the origin of the cumulative synaptic effect in vertical PEDOT:PSS OECTs by highlighting the temporal decoupling between ionic and electronic recovery processes. During a presynaptic spike group, large polycations (pDADMAC) penetrate the PEDOT:PSS channel and electrostatically interact with PSS^−^ chains, inducing dedoping of PEDOT and suppressing channel conductance. Upon removal of the gate bias, small Cl^−^ anions can rapidly redistribute to partially neutralize the accumulated polycations, whereas the back‐diffusion of the bulky polycations is kinetically hindered by the top drain structure. As a result, a fraction of polycations remains trapped beneath the drain, leaving portions of the PEDOT channel in a partially de‐doped state even though the macroscopic postsynaptic current appears to recover. This residual ionic imbalance lowers the effective conductance baseline for subsequent spike groups, leading to accumulated inhibition when stimuli are applied at short intervals. With longer inter‐spike intervals, gradual diffusion and neutralization of the trapped polycations erase this hidden ionic memory, restoring short‐term depression behavior. These observations demonstrate that the apparent long‐term‐like memory in polymer OECTs arises from incomplete ionic relaxation and spatial confinement of ions, rather than from permanent electronic or structural changes.

Li et al. reported a stretchable OECT array with synaptic functionality fabricated on a microstructured elastomeric substrate using a multimaterial 3D printing strategy [180]. In this work, all functional layers of the synaptic transistor, including stretchable source and drain electrodes, a PEDOT:PSS organic channel, a solid state electrolyte, and insulating layers, were sequentially patterned via direct ink writing using intrinsically stretchable inks (Figure 7d). Silver flake elastomer composites were employed for stretchable interconnects, while PEDOT:PSS served as the mixed ionic electronic conducting channel, enabling volumetric electrochemical modulation. The electrolyte layer, formulated from an ionically conductive polymer matrix, facilitates efficient ion transport under gate bias while maintaining mechanical compliance. To ensure mechanical robustness under deformation, the devices were implemented on a microstructured elastomeric substrate, which redistributes applied strain and suppresses crack formation in the printed functional layers. This structural design, together with the intrinsic stretchability of the printed materials, allows the OECT array to sustain large tensile strains while preserving stable electrochemical gating behavior (Figure 7e). Despite mechanical deformation, the devices exhibit characteristic synaptic features, including gradual conductance modulation and history‐dependent responses under pulsed gate operation, confirming that ion penetration and electrochemical doping and dedoping of the PEDOT:PSS channel remain effective in the stretchable configuration (Figure 7f). From a materials and manufacturing perspective, this work demonstrates how multimaterial 3D printing can serve as a powerful platform for neuromorphic electronics beyond rigid and planar substrates. By combining stretchable conductors, organic mixed‐conducting channels, and polymer electrolytes in a single additive process, the approach bypasses the limitations of conventional microfabrication and enables customizable, large‐area, and mechanically compliant synaptic arrays. Such process versatility is particularly relevant for wearable and biointegrated neuromorphic systems, where mechanical adaptability and intimate coupling with soft biological tissues are as critical as electrical performance.

As summarized in Table 1, these channel materials exhibit distinct operational characteristics in terms of active ion species, electrolyte configuration, conductance range, operating voltage window, and weight update symmetry [12, 51, 158, 168, 181, 182, 183, 184, 185]. For example, TMO‐based devices typically operate through Li^+^ or H^+^ intercalation within solid or liquid electrolytes, showing moderate conductance modulation and relatively stable asymmetry ratios under controlled voltage ranges. In contrast, 2D material systems demonstrate wider operation windows and tunable conductance levels depending on the interlayer ionic environment and electrolyte type, reflecting their interface‐dominated ion modulation. Organic mixed conductors, such as PEDOT‐based OECTs, generally provide significantly higher conductance and transconductance values at low operating voltages due to volumetric electrochemical doping, although their symmetry and retention characteristics depend strongly on ion mobility and trapping dynamics.

**TABLE 1:** Comparison of material systems and electrical characteristics of battery‐ion‐based synaptic transistors.

Channel material/type	Electrolyte/type	Active ion	Conductance	Operation voltage range	Asymmetry ratio	Ref
WO_3_/Graphene (TMO)	LiClO_4_ (Solid)	Li^+^	0.89–23 µS	0 to 4	0.26	[158]
LiCoO_2_ (TMO)	LiON (Solid)	Li^+^	257–268 µS	−4 to 0	0.17	[12]
MoO_3_ (TMO)	Ionic liquid (liquid)	H^+^	95 nS	−1 to 1	0.2	[181]
MoS_2_ (Two‐dimensional)	Lithium silicate (Solid)	Li^+^	1.5–8.5 nS	−6 to 12	0.32	[182]
MoS_2_ (Two‐dimensional)	LiCoO_2_ (Solid)	Li^+^	0.15–0.28 nS	−5 to 5	0.24	[183]
MoSe_2_ (Two‐dimensional)	N/A	K^+^	150–650 nS	−1 to 1	0.45	[184]
PEDOT:PSS (Organic)	KCl (Ion‐gel)	H^+^	∼ 750 µS	−0.3 to 0.5	N/A	[168]
PEDOT:Tos/PTHF (Organic)	NaCl (Ion‐gel)	Na^+^	2.4–3.1 mS	−0.8 to 0.8	N/A	[51]
Pg2T‐T (Organic)	pDADMAC (Ion‐gel)	Cl^−^	0.1–1.5 mS	−0.2 to 0.8	0.2	[185]

## 4 Applications of Battery‐Ion‐Based Neuromorphic

Battery‐inspired synaptic transistors are not only of fundamental interest as biologically faithful artificial synapses, but also offer distinctive advantages for a wide range of neuromorphic applications. From an application perspective, the electrochemical nature of battery‐inspired synaptic transistors also enables seamless coupling between sensing, memory, and computation. In this section, we review representative application domains of battery‐ionbased neuromorphic devices, focusing on their use in neuromorphic computing architectures and neuromorphic biosensing platforms.

### 4.1 Neuromorphic Computing

Neuromorphic computing aims to implement learning and information processing paradigms inspired by biological neural networks, where computation emerges from the collective dynamics of interconnected synaptic elements [186, 187, 188, 189]. Within this context, battery‐ion‐based synaptic transistors offer unique advantages for hardware implementation of neuromorphic computing, as their conductance states are governed by accumulated ionic distributions rather than instantaneous electronic switching. Such ion‐defined states naturally provide analog tunability, history dependence, and temporal dynamics, which are essential for both artificial neural network (ANN) implementations and emerging computing paradigms such as reservoir computing. Recent studies have increasingly explored reservoir sensing and neuromorphic sensing architectures that integrate sensing and information processing within a unified framework [190, 191]. As a result, the distinction between neuromorphic sensing and neuromorphic computing can sometimes be ambiguous. However, this review focuses on the role of the electrochemical synaptic device itself.

In ANN‐oriented neuromorphic hardware, precise and linear modulation of synaptic weights is a critical requirement for achieving high learning accuracy and stable convergence during training [192, 193, 194]. Battery‐inspired synaptic transistors based on TMOs are particularly attractive in this regard. These materials accommodate reversible ion insertion and extraction within their crystal lattices, enabling continuous control of the electronic structure and carrier density as a function of ionic concentration [42, 76]. As a result, synaptic weight updates can be tuned in a highly linear and analog manner by controlling the amplitude, duration, or number of programming pulses. Compared to filamentary or polarization‐based devices, where conductance changes are often abrupt or stochastic, ion intercalation‐driven modulation provides smoother weight evolution and reduced update noise.

**FIGURE 8:**
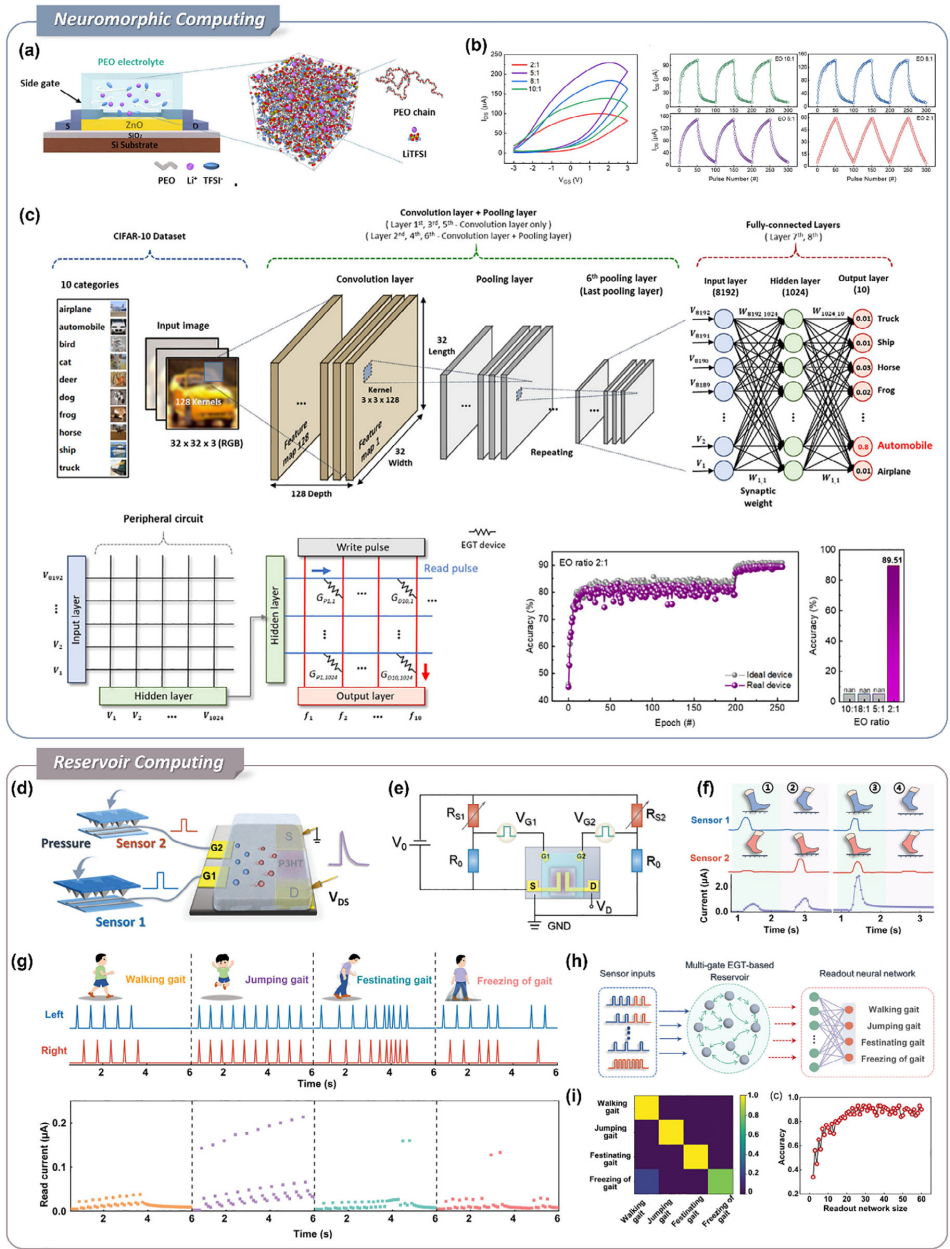
System‐level implementation of battery‐inspired synaptic transistors for neuromorphic and reservoir computing. (a–c) Neuromorphic computing: (a) Schematic of the side‐gated Li‐based EGT and molecular representation of the PEO/LiTFSI ion network governing conductance modulation. (b) Transfer characteristics and gradual analog conductance updates under consecutive voltage pulses. (c) Application of the device as programmable synaptic weights in a neural network for image classification, showing stable multilevel modulation and training accuracy. Reproduced with permission [195]. 2025, John Wiley and Sons. (d‐i) Reservoir computing: (d) Conceptual illustration of reservoir computing using a multi‐gate EGT platform. (e) Equivalent circuit of the multi‐gate EGT highlighting gate‐channel ionic coupling. (f) Transient current responses under independent sensor inputs, showing nonlinear temporal dynamics and short‐term memory effects. (g) Preprocessed temporal signals corresponding to different gait patterns and their reservoir responses. (h) Schematic of the reservoir computing framework combining the EGT reservoir and a readout neural network. (i) Confusion matrix and classification accuracy as a function of readout network size. Reproduced with permission [197]. 2023, The American Association for the Advancement of Science.

In 2025, Lee et al. provided a representative example illustrating how Li^+^‐ion‐mediated conductance modulation in oxide semiconductor channels can be exploited for neuromorphic computing applications [195]. By employing a ZnO channel in an EGT configuration with a PEO/LiTFSI electrolyte, the study highlights the feasibility of emulating key synaptic functionalities, including analog weight update, synaptic plasticity, and long‐term memory behavior, through controlled ionic motion at the channel interface (Figure 8a). In this system, the ratio between ethylene oxide (EO) units and Li^+^ ions in the electrolyte serves as a key parameter governing ionic transport and device operation. Adjusting the EO:Li^+^ ratio alters both ion density and mobility, resulting in distinct hysteretic transfer characteristics and memory windows (Figure 8b, left). The analog conductance modulation under pulsed gate bias further illustrates how ion dynamics can be tailored to achieve gradual and linear weight updates (Figure 8b, right). In particular, electrolyte compositions with higher Li^+^ concentration tend to promote hopping‐dominated ion transport, which suppresses abrupt conductance changes and favors smoother conductance evolution. Such characteristics are advantageous for ANNs by enabling linear and symmetric weight updates, which are essential for accurate learning and inference.

Beyond individual device characteristics, this work further illustrates how Li^+^‐based EGT arrays can be extended to system‐level neuromorphic computing tasks. As depicted in Figure 8c, arrays of EGTs are configured to emulate synaptic connections, where the continuously tunable conductance states of each transistor represent synaptic weights in a neural network. Notably, devices operated with an EO:Li^+^ ratio of 2:1, which exhibit the most linear conductance modulation, achieve the highest image classification accuracy of 89.51% in convolutional neural networks (CNN)‐based recognition of the Canadian Institute for Advanced Research (CIFAR)‐10 dataset. Simulation results indicate that the EGTs with an EO:Li^+^ ratio of 2:1 achieve the highest classification accuracy of 89.51%, owing to their superior linearity (nonlinearity of potentiation, NLP = 0.42; nonlinearity of depression NLD = −0.5) and large dynamic range (DR = 11.64). These results highlight the critical importance of controllable and highly linear conductance modulation in battery‐ion‐based synaptic devices, underscoring their significance for achieving high‐performance and energy‐efficient neuromorphic computing systems.

In addition to ANN implementations, battery‐ion‐based synaptic devices are also well aligned with reservoir computing, which exploits the rich internal dynamics of a nonlinear physical system rather than explicit weight training within the reservoir itself [196]. In reservoir computing, the diversity and fading memory of internal states are key factors determining computational performance. In electrochemical synaptic transistors, different ionic configurations established by prior stimuli give rise to a multitude of conductance states and relaxation pathways, effectively creating a high‐dimensional state space within a single device or device network. Because ionic redistribution and relaxation occur over multiple timescales, these devices inherently exhibit short‐term memory and nonlinear dynamics, which are essential for reservoir operation. The dependence of conductance on ionic history allows reservoir states to be encoded and evolved through ion‐mediated processes, enabling temporal signal processing and dynamic pattern recognition without the need for precise weight updates within the reservoir.

The ability to process multiple input streams while retaining temporal information is particularly important for reservoir computing systems. To address this requirement, the multi‐gate EGT with a P3HT channel was utilized as an ion‐mediated reservoir computing platform for multichannel near‐sensor computing applications [197]. In this architecture, the multi‐gate EGT functions as a physical reservoir that simultaneously integrates multichannel input acquisition and temporal feature extraction within a single device. By exploiting ion‐mediated conductance dynamics rather than explicit weight programming, the system illustrates how reservoir computing can be realized with high energy efficiency and reduced circuit complexity. As illustrated in Figure 8d,e, wearable pressure sensors are serially connected to the EGT through a voltage divider circuit, enabling gait‐related signals to be directly coupled into the ionic gating process. Two independent gates are used to represent bilateral inputs during bipedal walking, allowing pressure signals from both feet to be processed in parallel. This multi‐gate configuration increases the dimensionality of the reservoir input space and enhances the richness of internal ionic dynamics, which is a key requirement for effective reservoir computing.

To assess the potential of reservoir computing for Parkinson's disease diagnosis through gait pattern analysis, four representative gait patterns were selected: normal walking, jumping gait, festinating shuffling gait, and freezing of gait; the latter two being characteristic symptoms associated with Parkinson's disease. As shown in Figure 8f, these gait patterns exhibit distinct temporal pressure profiles. Specifically, the sensing inputs are obtained via external pressure sensors and converted into electrical signals, which are subsequently applied to the multi‐gate EGT device. When applied to the multi‐gate synaptic device, the four gait patterns induce different conductance trajectories that are stored as reservoir states (Figure 8g). This behavior indicates that the ion‐based reservoir effectively transforms raw sensor inputs into compact, history‐dependent states that encode salient temporal features of gait, thereby reducing the amount of data required for subsequent processing. The encoded reservoir states are subsequently processed by a simple readout layer consisting of a 60 × 4 neural network, where 60 reservoir states serve as inputs and four output neurons correspond to the different gait classes (Figure 8h). Importantly, only the readout layer is trained, while the internal reservoir dynamics remain unmodified, consistent with the reservoir computing paradigm. Using this approach, a gait classification accuracy of 93% is achieved (Figure 8i), demonstrating that ion‐mediated reservoir states can support reliable pattern recognition without complex weight training or extensive digital preprocessing. This work is an example showing how the slow, nonlinear, and history‐dependent motion of ions in electrolyte‐gated devices provides a natural physical basis for reservoir computing. By embedding sensing, memory, and computation directly into a single ion‐based device, multi‐gate EGT reservoirs enable efficient near‐sensor processing of spatiotemporal signals.

### 4.2 Neuromorphic Biosensors

Neuromorphic biosensors aim to bridge biological signals and artificial intelligence by directly interfacing with the ionic nature of living systems [175, 198]. In this context, battery‐ion‐based neuromorphic devices provide a fundamental advantage over conventional electron‐driven transistors, as their operation is governed by the migration, accumulation, and relaxation of mobile ions, which closely resemble ion transport in biological tissues and neural systems. This intrinsic bio‐responsiveness enables battery‐ion‐based synaptic devices to interact naturally with physiological signals, such as ionic fluxes, electrochemical potentials, and biomolecular binding events, without requiring complex signal transduction or amplification stages [199, 200]. Beyond simple signal detection, the slow and history‐dependent ion dynamics endow these devices with memory, nonlinearity, and rich temporal responses, allowing biological inputs to be directly encoded into synaptic states. Such characteristics are particularly attractive for neuromorphic biosensing, where sensing, preprocessing, and learning can be merged at the device level.

**FIGURE 9:**
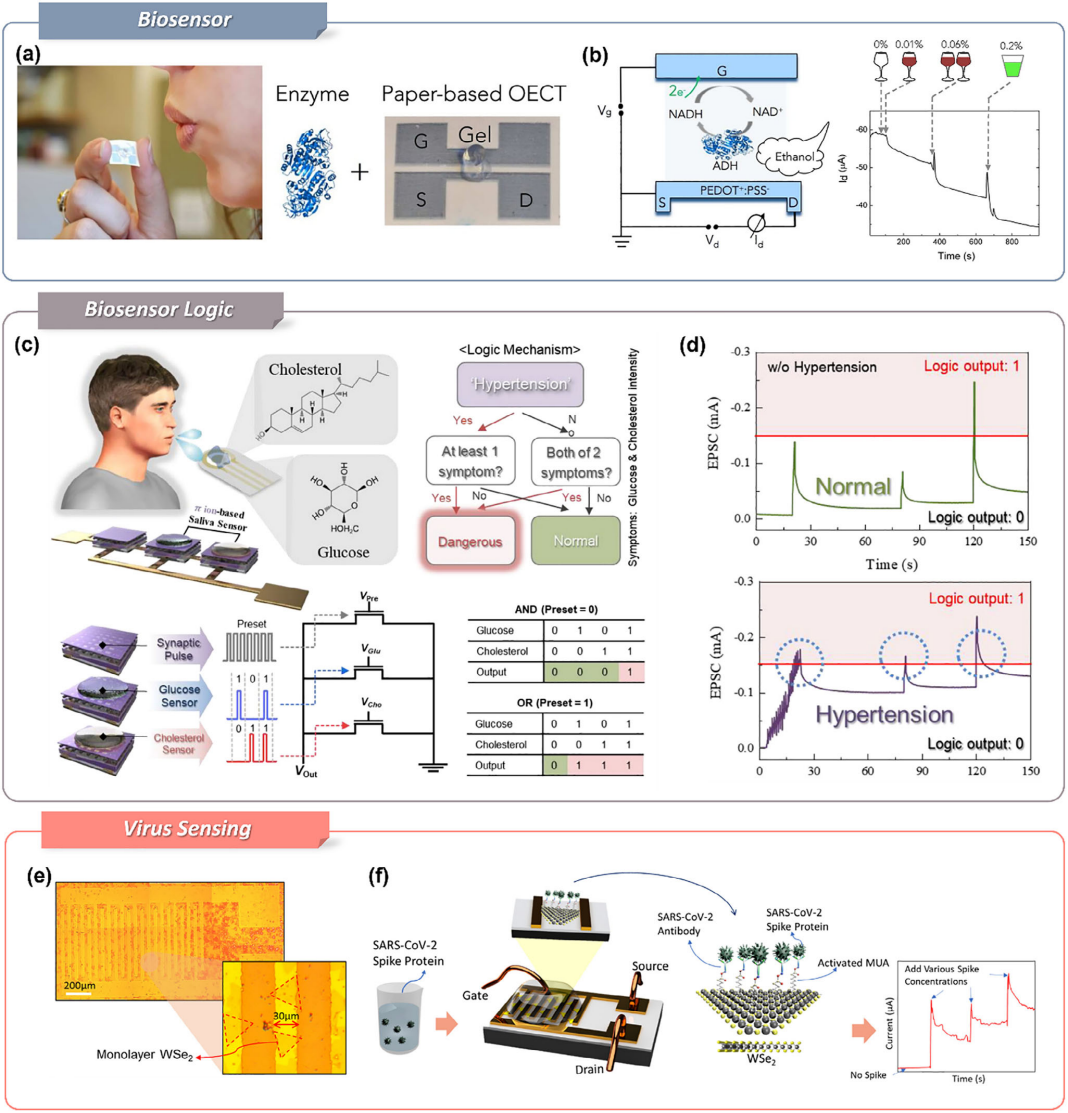
Comprehensive neuromorphic biosensor applications of ion‐mediated synaptic devices. (a, b) Biosensing platforms: OECT‐based alcohol breathalyzer: Implementation of a disposable, paper‐based OECT using PEDOT:PSS. By immobilizing alcohol dehydrogenase (ADH) and its cofactor NAD^+^ in an electrolyte gel, the device detects ethanol in the breath equivalent to a Blood Alcohol Content (BAC) of 0.01%–0.2%. Reproduced with permission [201]. 2016, Springer Nature. (c, d) Biosensor logic and diagnostics: Schematic of a wearable saliva sensor system that processes glucose and cholesterol levels through synaptic logic gates (AND/OR). Reproduced with permission [202]. 2025, John Wiley and Sons. (e, f) Nanoscale sensing interfaces: Optical microscope images and operational schematics of monolayer WSe_2_ sensors tailored for specific bio‐recognition, showcasing the scalability and versatility of 2D material‐based neuromorphic interfaces. Reproduced with permission [203]. 2021, American Chemical Society.

As a relevant study, in 2016, Bihar et al. reported an alcohol‐sensing paper‐based OECT that exemplifies how ion‐mediated device operation can be directly coupled to biochemical reactions for neuromorphic biosensing (Figure 9a) [201]. In this work, a PEDOT:PSS‐based OECT was functionalized with alcohol dehydrogenase (ADH) and its cofactor NAD^+^, which were immobilized within an electrolyte gel bridging the gate and channel. Upon exposure to ethanol, enzymatic oxidation reactions convert NAD^+^ to NADH, generating ionic and electronic charge redistribution within the electrolyte (Figure 9b top). As shown in Figure 9b (bottom), this process effectively modulates the ionic environment at the channel/electrolyte interface, leading to a gradual decrease in channel conductance through electrochemical dedoping of PEDOT:PSS. From a neuromorphic perspective, the sensing mechanism is not limited to instantaneous signal transduction but inherently relies on ion accumulation, relaxation, and diffusion processes that unfold over time. The ethanol‐induced ionic redistribution introduces temporal integration and memory effects into the device response, allowing biochemical stimuli to be encoded as history‐dependent conductance states rather than transient electrical signals. Such behavior highlights a key advantage of battery‐ion and ion‐driven neuromorphic devices; biological recognition events are naturally mapped onto synaptic‐like state changes without external amplification or complex signal conditioning. In this context, the OECT breathalyzer can be viewed not merely as a chemical sensor, but as an early example of a neuromorphic biosensor in which ionic processes simultaneously enable sensing, state modulation, and analog information encoding.

As another report with an emphasis on neuromorphic biosensor applications, in 2025, Woo et al. further extended battery‐ion‐based neuromorphic devices toward logic‐enabled biosensing, demonstrating how ionic dynamics can be exploited to move beyond binary detection toward decision‐oriented sensing (Figure 9c) [202]. In contrast to conventional biosensors that independently detect biomarkers and output digital on/off signals, their neuromorphic sensing strategy integrates multiple biochemical inputs and evaluates their combined significance through logic operations implemented at the device level. As illustrated in the bio‐sensor logic framework, cholesterol and glucose signals are simultaneously introduced to the ion‐responsive synaptic system, where their presence modulates ionic redistribution within the reinforced‐ion film. By configuring AND and OR logic operations, the device evaluates the co‐occurrence or dominance of these biomarkers rather than treating them as isolated signals. This approach reflects a clinically relevant diagnostic logic, as hypertension risk is more accurately inferred from the combined elevation of multiple biomarkers rather than a single parameter. It is noted that the neuromorphic nature of the sensing scheme enables temporal filtering through ion‐mediated memory effects. Instead of assigning a hypertensive state based on a single sensing event, the system recognizes hypertension only when the combined biomarker logic is repeatedly reinforced over time (Figure 9d). The slow accumulation and relaxation of ions introduce a form of temporal integration, allowing sporadic or noisy inputs to decay naturally while persistent biochemical patterns are consolidated into stable conductance states.

As an example presenting a virus detection, Fathi‐Hafshejani et al. reported a 2D material‐based field‐effect transistor biosensor for the detection of the SARS‐CoV‐2 virus, highlighting how ion‐sensitive transduction mechanisms can be leveraged for highly sensitive and selective viral sensing (Figure 9e) [203]. In this platform, specific binding between viral antigens and functionalized receptor layers at the transistor surface perturbs the local ionic and electrostatic environment, leading to measurable modulation of the channel conductance. As shown in Figure 9f, the device does not rely solely on instantaneous electronic responses but instead converts biomolecular recognition events into sustained conductance changes through interfacial charge redistribution and ion‐related screening effects. From a neuromorphic biosensing perspective, such ion‐coupled transduction provides a direct pathway for encoding viral binding events into analog and persistent device states. The gradual conductance evolution induced by biomolecular interactions reflects accumulated ionic perturbations at the sensing interface, enabling discrimination between target and non‐target species with enhanced signal robustness. This behavior is particularly relevant for infectious disease diagnostics, where reliable detection under low viral load and noisy biological environments is critical. By exploiting ion‐mediated signal amplification and retention, the reported approach illustrates how biosensing and memory can be inherently co‐integrated at the device level.

Taken together with ion‐based alcohol sensing and logic‐enabled metabolic diagnostics, these examples show the broader implications of battery‐ion and ion‐driven neuromorphic biosensors. By utilizing ionic motion as a shared physical mechanism for sensing, memory, and computation, such devices move beyond conventional threshold‐based detection toward adaptive and context‐aware biosensing. The ability to encode biochemical information into synaptic‐like states, perform logic evaluation, and suppress spurious responses through temporal integration highlights the potential of ion‐based neuromorphic platforms for next‐generation healthcare monitoring, where reliability, energy efficiency, and biological compatibility are simultaneously required.

## 5 Challenges and Perspectives

**FIGURE 10:**
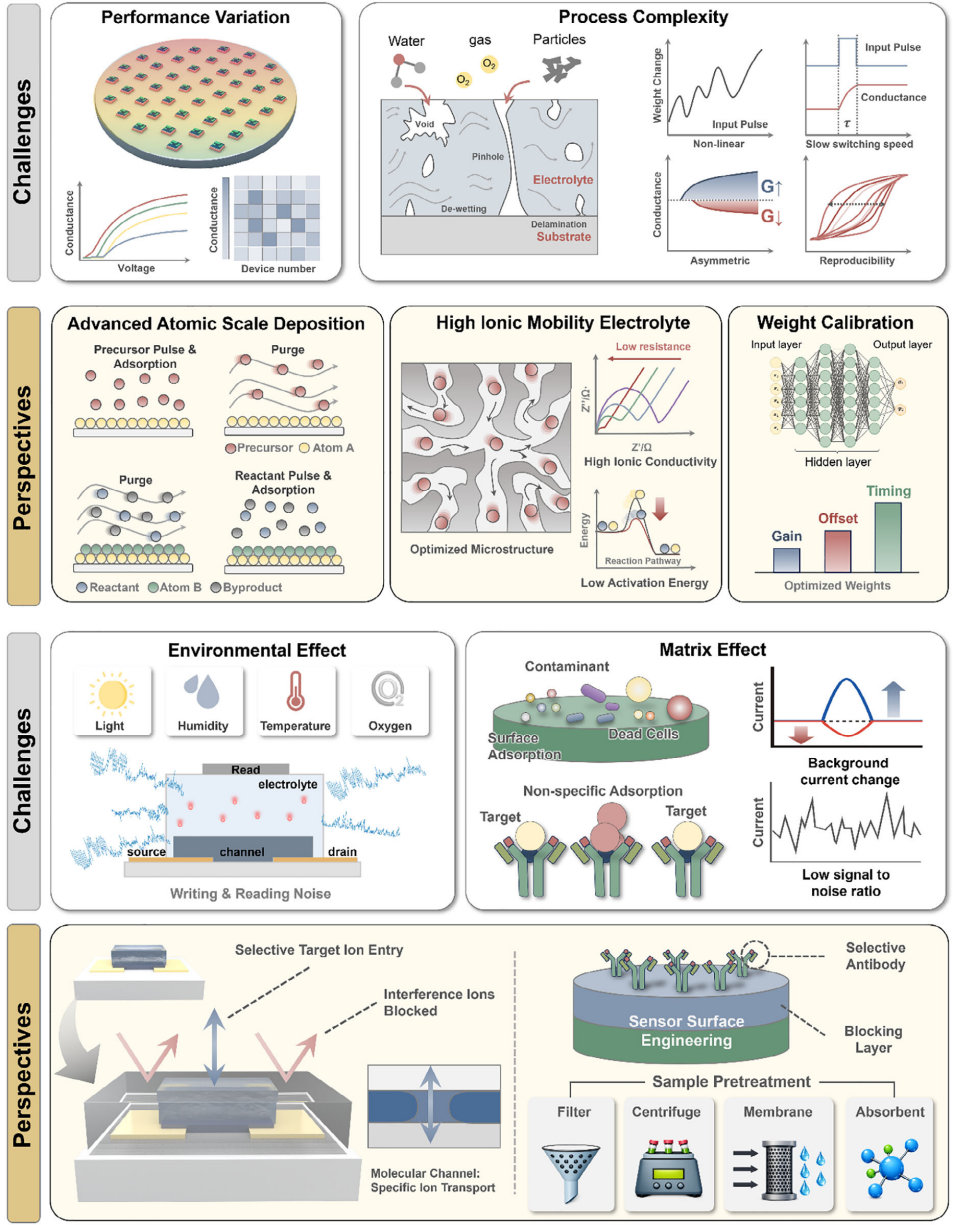
Comprehensive overview of challenges and future perspectives for the practical application of neuromorphic electrochemical devices. (Top) Challenges in operational robustness include wafer‐scale performance variation, process complexities at the electrolyte‐channel interface, and non‐ideal synaptic updates. Promising perspectives to overcome these issues include advanced atomic‐scale deposition techniques, the development of high ionic mobility electrolytes with optimized microstructures, and algorithmic weight calibration. (Bottom) Challenges induced by external factors highlight environmental effects and matrix effects, which refer to signal interferences from non‐target components in complex samples, thereby lowering the signal‐to‐noise ratio due to non‐specific adsorption and surface contaminants. Future perspectives for addressing these issues involve specific ion transport through engineered molecular channels, sensor surface engineering, and systematic sample pretreatment procedures.

Electrochemical battery‐inspired synaptic devices have enabled meaningful advances in neuromorphic electronics by offering diverse device‐level functionalities, ranging from symmetric synaptic plasticity and dynamic weight modulation to efficient ion‐coupled signal processing. These demonstrations provide the potential of versatile neuromorphic hardware, enabled by tailoring ion transport dynamics, electrolyte properties, and interfacial electrochemistry. However, despite considerable progress at the individual device level, realizing system‐level neuromorphic computing architectures and practical biosensing platforms that are scalable, operationally robust, and environmentally stable still faces several critical challenges. Addressing these critical challenges requires a thorough evaluation of operational robustness at the device level and vulnerabilities to external factors. Figure 10 comprehensively illustrates these specific hurdles alongside the technological directions and future perspectives required to overcome them.

Challenges in operational robustness include array‐level non‐uniformities, integration difficulties, and non‐ideal switching dynamics [28, 204]. Scaling these devices to large‐area arrays inevitably introduces significant performance variation. Unlike purely electrostatic devices, this variation is uniquely exacerbated in electrochemical systems because their synaptic modulation fundamentally relies on physical ion mass transfer and interfacial redox reactions [205, 206, 207]. Even minute spatial non‐uniformities in electrolyte morphology or interfacial defect densities across a substrate drastically alter localized ion diffusion pathways and reaction kinetics, which severely degrades the reliability of neural network mapping. Parallel to this, device fabrication involves severe process complexities. To be specific, the widespread use of soft ion‐conducting materials, such as liquid electrolytes and ion‐gels, presents substantial integration challenges due to their fluidic nature and incompatibility with conventional patterning processes [105, 172]. The electrolyte‐channel interface is particularly susceptible to structural and physical defects during integration, frequently resulting in the formation of voids, pinholes, dewetting, and delamination. Furthermore, the intrinsic electrochemical processes themselves often yield non‐ideal synaptic characteristics, manifesting as non‐linear and asymmetric conductance updates, slow switching speeds, and limited cycle‐to‐cycle reproducibility.

For array integration, device‐to‐device and spatial non‐uniformity in ion‐mediated conductance modulation is an important reliability concern. Variations in electrolyte morphology, interfacial defect density, film thickness, and local ion‐transport pathways can affect switching kinetics, memory window, conductance‐update linearity, and retention characteristics, thereby limiting the reproducibility of synaptic weight mapping. Recent studies suggest that such variations can be mitigated by improving the uniformity of electrolyte and active‐layer formation using vacuum‐compatible thin‐film deposition processes [208, 209, 210, 211, 212]. Solid or quasi‐solid electrolytes, interfacial stabilization, and optimized pulse operation can further improve the stability of ion‐mediated conductance modulation [82, 121, 140, 185, 213, 214, 215, 216, 217]. Remaining non‐uniformity and non‐ideal conductance updates may also be compensated through array‐level calibration or write‐verify schemes [218, 219, 220]. These considerations indicate that reliable array implementation requires coordinated control of materials, interfaces, device operation, and circuit‐level compensation.

Overcoming these fundamental hardware limitations necessitates a comprehensive approach, moving from structural engineering to system‐level compensation. To suppress interfacial defects and spatial non‐uniformities, transitioning toward advanced atomic‐scale deposition techniques offers a promising pathway. Such precise deposition strategies could enable highly conformal, defect‐free interfaces, fundamentally suppress the formation of voids and pinholes while ensure uniform ion transport across large‐area arrays. Furthermore, replacing fluidic soft materials with rigid, solid‐state alternatives can resolve integration incompatibilities, solid electrolytes traditionally suffer from limited ion transport. Thus, engineering these solid electrolytes to achieve high ionic mobility becomes a critical requirement. By optimizing their microstructures to lower the activation energy for ion migration, it may be possible to secure both the structural integrity required for reliable fabrication and the fast, reproducible kinetics needed for efficient switching. Finally, while material optimizations can substantially refine device dynamics, completely eliminating intrinsic electrochemical non‐idealities remains challenging. In this regard, implementing algorithmic weight calibration provides a practical workaround. By dynamically adjusting network weights to compensate for the remaining non‐linear and asymmetric conductance updates, high‐precision neuromorphic training could be successfully realized despite the underlying hardware imperfections [221].

For these reasons, solid‐state electrolytes are promising for practical wearable and bio‐integrated neuromorphic systems because they can provide leakage‐free operation, material stability, process compatibility, and long‐term reliability [49, 222, 223, 224, 225, 226, 227]. These features are important not only for large‐area arrays requiring uniform film formation and high process yield, but also for wearable and bio‐interfaced devices that must maintain stable operation under mechanical deformation, humidity, and biological environments. However, their relatively limited ionic mobility compared with liquid or gel‐based electrolytes remains a key bottleneck, as reduced ionic redistribution under electrical bias can weaken ion‐mediated channel modulation and may result in increased operating voltages or longer pulse durations for effective synaptic weight updates [27, 66, 228, 229, 230, 231, 232]. Therefore, future efforts should focus on improving ion transport while preserving the inherent stability and manufacturability of solid‐state electrolytes. Because ionic conduction generally follows the Arrhenius law, ionic conductivity can be enhanced by increasing the density of mobile ions, promoting effective migration pathways, and reducing the activation energy for ion hopping [98, 99, 100, 101].

Beyond these intrinsic hardware characteristics, practical deployment is significantly hindered by external factors, especially when devices are integrated into complex environments. When exposed to complex operational environments, these devices exhibit extreme sensitivity to ambient fluctuations; variations in light, humidity, temperature, and oxygen introduce severe writing and reading noise into the system, compromising operational stability. More critically, when operating in complex biological fluids, these sensors suffer from matrix effects, which refer to signal interferences from non‐target components [198]. Background molecules and dead cells frequently cause non‐specific adsorption on the sensor surface, thereby drastically lowering the signal‐to‐noise ratio (SNR) and degrading sensing fidelity. Future perspectives for addressing these issues require a holistic, multi‐layered approach. At the device architecture level, engineered molecular channels can facilitate specific target ion transport while effectively blocking interferents. In parallel, rigorous surface engineering is imperative to suppress non‐specific binding on the active area. This involves the strategic integration of selective antibodies for high‐specificity recognition and blocking layers to minimize background interference. As the last note of outlook, integrating systematic sample pretreatment procedures, such as filtering and centrifugation, will be crucial to isolate target analytes and ensure high‐fidelity sensory inputs for accurate neuromorphic processing.

## 6 Conclusions

Battery‐inspired electrochemical synapses provide a conceptually consistent route to neuromorphic devices. Based on principles established in electrochemistry, including ionic transport, reversible redox reactions, and electrochemical state evolution, these devices naturally support gradual, accumulative, and time‐dependent conductance modulation. Such characteristics enable the emulation of key synaptic functions, including short‐ and long‐term plasticity, multilevel weight storage, and low‐voltage operation within a single device platform. Researchers have expanded the functionality of battery‐ion‐inspired synaptic devices by integrating diverse electrolyte types and channel materials, enabling tailored performance for neuromorphic computing and sensing applications.

From an electrochemical perspective, synaptic transistors based on diverse material systems share common governing factors related to ionic mobility, stability, and reversibility. This review has revisited and thereby discussed how electrochemical design choices shape synaptic performance and practical considerations relevant to real‐world implementation, including scalability, variability, and long‐term reliability. Relating electrochemical synaptic devices to established concepts in energy storage enables a coherent understanding of their capabilities and limitations, while clarifying pathways toward learning‐enabled information processing and adaptive sensing applications.

## Author Contributions

W. W. Lee, S. C. Jang, and W. Choi contributed equally to this work. W. W. Lee and S. C. Jang conceived the main concept of this review. W. W. Lee conducted the comprehensive literature search on battery‐inspired electrochemical synapses, organized the overall structure of the manuscript, and wrote the original draft. W. Choi assisted with literature review and visualization. J. Park assisted with the literature search. J. Hur, H. Yoo, and H. ‐S Kim supervised the entire project, provided critical feedback, and secured funding.

## Conflicts of Interest

The authors declare no conflicts of interest.

## Data Availability

The data that support the findings of this study are available from the corresponding author upon reasonable request.
